# Wnt-regulated lncRNA discovery enhanced by in vivo identification and CRISPRi functional validation

**DOI:** 10.1186/s13073-020-00788-5

**Published:** 2020-10-22

**Authors:** Shiyang Liu, Nathan Harmston, Trudy Lee Glaser, Yunka Wong, Zheng Zhong, Babita Madan, David M. Virshup, Enrico Petretto

**Affiliations:** 1 Program in Cancer and Stem Cell Biology, Duke-NUS Medical School, Singapore, Singapore; 2 Program in Cardiovascular and Metabolic Disorders, Duke-NUS Medical School, Singapore, Singapore; 3 Science Division, Yale-NUS College, Singapore, Singapore; 4 Department of Pediatrics, Duke University School of Medicine, Durham, North Carolina USA; 5 MRC London Institute of Medical Sciences, Imperial College London, London, UK

**Keywords:** Functional lncRNAs, Wnt signaling, Cancer, CRISPRi screen

## Abstract

**Background:**

Wnt signaling is an evolutionarily conserved developmental pathway that is frequently hyperactivated in cancer. While multiple protein-coding genes regulated by Wnt signaling are known, the functional lncRNAs regulated by Wnt signaling have not been systematically characterized.

**Methods:**

We comprehensively mapped Wnt-regulated lncRNAs from an orthotopic Wnt-addicted pancreatic cancer model and examined the response of lncRNAs to Wnt inhibition between in vivo and in vitro cancer models. We further annotated and characterized these Wnt-regulated lncRNAs using existing genomic classifications (using data from FANTOM5) in the context of Wnt signaling and inferred their role in cancer pathogenesis (using GWAS and expression data from the TCGA). To functionally validate Wnt-regulated lncRNAs, we performed CRISPRi screens to assess their role in cancer cell proliferation both in vivo and in vitro.

**Results:**

We identified 3633 lncRNAs, of which 1503 were regulated by Wnt signaling in an orthotopic Wnt-addicted pancreatic cancer model. These lncRNAs were much more sensitive to changes in Wnt signaling in xenografts than in cultured cells. Our analysis suggested that Wnt signaling inhibition could influence the co-expression relationship of Wnt-regulated lncRNAs and their eQTL-linked protein-coding genes. Wnt-regulated lncRNAs were also implicated in specific gene networks involved in distinct biological processes that contribute to the pathogenesis of cancers. Consistent with previous genome-wide lncRNA CRISPRi screens, around 1% (13/1503) of the Wnt-regulated lncRNAs were found to modify cancer cell growth in vitro. This included *CCAT1* and *LINC00263*, previously reported to regulate cancer growth. Using an in vivo CRISPRi screen, we doubled the discovery rate, identifying twice as many Wnt-regulated lncRNAs (25/1503) that had a functional effect on cancer cell growth.

**Conclusions:**

Our study demonstrates the value of studying lncRNA functions in vivo, provides a valuable resource of lncRNAs regulated by Wnt signaling, and establishes a framework for systematic discovery of functional lncRNAs.

## Background

Long noncoding RNAs (lncRNAs) play key roles in diverse biological processes, ranging from development, such as *XIST* for dosage compensation [1] and *H19* for imprinting [2], to different diseases including cancer [3]. lncRNAs have been shown to play important roles in fundamental biological signaling pathways regulated by P53, Notch, and TGF-β [4–6]. lncRNAs can contribute to the development of cancer through aberrant expression or mutation, altering their normal physiological functions in signaling pathways [7]. Advancements in transcriptomics have greatly expanded the number of lncRNAs annotated in the human genome [8, 9], but only a small fraction have been characterized at a functional level.

Wnt/β-catenin signaling is an important evolutionarily conserved signaling pathway that is crucial for embryonic development and tissue regeneration [10]. After Wnt ligands bind to Frizzled and other co-receptors on the cell surface, β-catenin is stabilized and translocates into the nucleus, where it interacts with TCF/LEF transcription factors in a context-dependent manner to regulate the expression of multiple protein-coding genes such as *MYC* and *AXIN2*. Dysregulation of Wnt signaling is found in multiple cancers. The most common mutations activating Wnt/β-catenin signaling occur in colorectal cancer, where truncations of APC cause abnormal stabilization of β-catenin and constitutive transcriptional activation [11–13]. A different class of mutations confers cancer dependency on Wnt ligands. For example, *RNF43* and *RPSO3* mutations cause increased abundance of Wnt receptors on the cell surface, making the cancer cells addicted to Wnt signaling [14–16]. RNF43 mutations are found in 5–10% of pancreatic cancers, while RPSO3 translocations are found in 10% of colorectal cancers [16–20].

Wnt addiction in cancer presents a therapeutic opportunity [21]. All Wnts require palmitoleation in the endoplasmic reticulum by the enzyme PORCN for their secretion and function [22]. Small molecule PORCN inhibitors block this modification and hence the activity of all Wnts. We and others have demonstrated that PORCN inhibitors such as ETC-159 can effectively suppress Wnt signaling and the growth of Wnt-addicted cancers in multiple preclinical models [14, 23, 24]. Due to its efficacy, the PORCN inhibitor ETC-159 has advanced to clinical trials [25]. ETC-159 is also a useful research tool to study Wnt-dependent genes. We found that more than 75% of the transcriptome responded to PORCN inhibition by ETC-159 in Wnt-addicted cancers, with significantly more genes changing in vivo than in vitro [24, 26]. Thus, PORCN inhibition is a powerful tool to study Wnt-regulated genes, and these Wnt-regulated genes are best studied in vivo in the presence of the appropriate microenvironment.

To date, only a few individual lncRNAs have been linked to Wnt signaling. For example, *MYU* (*VPS9D1-AS1*) is a target of Wnt/c-Myc signaling involved in the proliferation of colon cancer cells by upregulation of *CDK6* [27]. Wnt-regulated lncRNA *WiNTRLINC1* promotes proliferation and survival of colon cancer cells by regulating its genomic neighbor *ASCL2* through long-range chromosomal looping [28]. In addition, lncRNA *ASBEL* is a target of Wnt/β-catenin signaling. *ASBEL* forms a complex with TCF3, which is required for the tumorigenicity of colorectal cancer cells through transcriptional repression of ATF3 [29]. However, currently, there are no systematic studies on functional lncRNAs regulated by Wnt signaling in vivo. Here, we comprehensively mapped Wnt-regulated lncRNAs from an orthotopic Wnt-addicted pancreatic cancer model and determined their wider roles in other cancers. To functionally validate the Wnt-regulated lncRNAs, we performed CRISPRi screens both in vitro and in vivo. Notably, we found multiple Wnt-regulated lncRNAs that had functional effects on cancer cell growth only in a xenograft model, demonstrating the value of studying lncRNA functions in vivo. This study provides a valuable resource of functional lncRNAs regulated by Wnt signaling. It also establishes a framework that can be broadly adapted for systematic discovery and functional annotation and validation of lncRNAs in vivo.

## Methods

### De novo lncRNA discovery

The polyA+ RNA-seq dataset contains the transcriptional response to PORCN inhibitor ETC-159 treatment at seven time points (0, 3, 8, 16, 32, 56, and 168 h) using an orthotopic model of *RNF43*-mutant pancreatic adenocarcinoma (HPAF-II). The data was previously published under accession number GSE118041 [26]. RNA-seq reads were assessed for quality with FASTQC. Reads originating from mouse genome (mm10) were removed with Xenome [30]. All the reads among replicates from each time point were pooled to achieve deep coverage for novel lncRNA discovery. Each time point generated between 160 million to 237 million reads. The reads were aligned to hg38 (Ensembl version 79) using TopHat v2.0.10 [31]. De novo transcriptome assembly was performed separately for each time point with Cufflinks v2.1.1 [32]. Transcriptome assemblies at each time point were merged and compared with Ensembl build 79 as reference, using Cuffmerge. The novel transcripts were selected using Cuffcompare class code for novel intergenic and novel antisense transcripts. All the novel transcripts were then merged with Ensembl build 79 to establish a full reference transcriptome. RNA-seq reads from each sample were also individually aligned to hg38 (Ensembl version 79) using TopHat v2.0.10 [31]. Gene-level read counts for each sample were computed with HTSeq 0.6.0 [33], which were then converted to gene expression in Transcripts per Million (TPM). To identify putative novel lncRNAs transcripts, the novel transcripts were filtered using the following criteria: length longer than 200 bp and estimation to be non-protein coding based on three methods: CPAT with threshold less than 0.364 [34], CPC with threshold less than 0 [35], and Slncky defined as “lncRNA” [36]. Known lncRNAs from Ensembl build 79 were obtained based on their transcript biotype: “lincRNA,” “antisense,” “sense_intronic,” and “sense_overlapping”. All the genes were also filtered based on their expression to make sure that the median expression level of each gene at every time point had TPM > 1. This analysis yielded 16,160 genes, including 12,527 protein-coding genes, 2846 annotated lncRNAs, and 787 novel lncRNAs that were expressed in *RNF43*-mutant pancreatic adenocarcinoma (HPAF-II).

### Identification of Wnt-regulated lncRNAs

To identify genes regulated by Wnt signaling, DESeq2 [37] was used to perform differential expression analysis on 16,160 genes across time points by likelihood ratio test (LRT). Adjusted *P* value < 0.05 was used to select genes significantly responded to Wnt inhibition across time points. This led to 10,554 Wnt-regulated genes, including 9051 protein-coding genes and 1503 lncRNAs (1178 annotated lncRNAs and 325 novel lncRNAs, Additional file 2: Table S1).

### Comparison of lncRNAs response to Wnt inhibition across models

Two RNA-seq datasets contain transcriptional response of in vitro model (48 h ETC and 48 h Veh) and subcutaneous model (0 h and 56 h) of *RNF43*-mutant pancreatic adenocarcinoma (HPAF-II) to PORCN inhibitor ETC-159 treatment. The data was previously published under accession number GSE118190 and GSE118179, respectively [26]. RNA-seq reads from these datasets were assessed for quality with FASTQC (https://www.bioinformatics.babraham.ac.uk/projects/fastqc/). Reads originating from mouse genome (mm10) were removed with Xenome [30] and aligned to hg38 (Ensembl version 79) using TopHat v2.0.10 [31] for each sample. Gene-level read counts were computed with HTSeq 0.6.0 [33]. DESeq2 [37] was used to perform differential gene expression analysis on 16,160 genes between the time points with Wald test for each of the models, namely in vitro model (48 h ETC and 48 h Veh), subcutaneous model (0 h and 56 h), and orthotopic model (0 h and 56 h). An adjusted *P* value < 0.1 was used to select genes that significantly responded to Wnt inhibition between the two time points.

### Wnt-regulated lncRNA co-expression with protein-coding genes (PCGs)

The degree of co-expression between Wnt-regulated lncRNAs and either all PCGs or their nearest PCG in response to Wnt inhibition in the orthotopic HPAF-II cancer model was calculated by cor function (Spearman correlation) in R. The TAD data from the PANC-1 cell line mapped to hg38 was downloaded from the 3D Genome Browser [38]. The Wnt-regulated lncRNA and nearest PCG pair was classified into two groups, the pair in the same TAD versus the pair in different TADs based on the PANC-1 TAD information. The correlation distributions between the two groups were tested for difference by the nonparametric two-sample Mann–Whitney *U* test using the R function wilcox.test.

### Analysis of TCGA dataset

HTSeq-Counts data of all the TCGA cancers were downloaded from the UCSC Xena platform [39]. Genes with less than 10 reads mapped across the samples within each cancer type were removed. The gene read count tables were filtered to retain the tumor-normal sample pairs for each cancer type. PCA analysis was performed to select the cancer types with a clear separation between the tumor and normal samples. The gene read count and dispersion distribution were estimated and used for statistical power estimation using RnaSeqSampleSize package [40]. The statistical power was estimated with two sets of parameters: using different FDR (1%, 5% and 10%) while keeping the minimal fold change between two groups at 2; using different minimal fold change between two groups (1.5, 2, 2.5) while keeping the FDR at 5%. Differential expression analysis between the paired tumor-normal samples for each cancer type was performed using DESeq2 [37]. An adjusted *P* value (FDR) < 0.05 was used to select genes significantly differentially expressed between tumor and normal sample. Sixty-eight significantly upregulated and 10 significantly downregulated Wnt-regulated lncRNAs were found in pancreatic cancer (Additional file 3: Table S2). Based on the statistical power estimation, cancer types with less than 5 tumor-normal pairs had low statistical power of finding significantly differentially expressed genes between tumor and paired normal samples (Additional file 1: Fig. S2A), so they were excluded from the downstream analysis except for pancreatic cancer (PAAD), due to its relevance and potential interest to the study. This yielded 15 cancer types from the TCGA dataset.

### Integrative analysis of FANTOM5 dataset

Wnt-regulated lncRNAs were mapped to FANTOM5 lncRNA annotations as follows: (1) If the lncRNA was annotated with the same Ensembl Gene ID in FANTOM5, it is considered the same lncRNA. (2) The remaining lncRNAs were overlapped with FANTOM5 lncRNA assembly (hg38) to identify the corresponding FANTOM5 CAT_geneID. Among the 1503 Wnt-regulated lncRNAs, 1073 were also annotated in FANTOM5 and 430 were novel previously unannotated lncRNAs. The eQTL-linked lncRNA protein-coding gene (PCG) pairs for these 1073 annotated Wnt-regulated lncRNAs were extracted from FANTOM5 annotation eQTL_linked_lncRNA_mRNA_pair [8]. This yielded 1486 lncRNA-PCG mRNA pairs linked by eQTL SNPs involving 602 Wnt-regulated lncRNAs (Fig. 2a and Additional file 4: Table S3). The gene expression profiles of all the pairs in 1829 FANTOM5 samples were downloaded from the expression atlas FANTOM_CAT.expression_atlas.gene.lv3_robust.rle_cpm curated by FANTOM5 [8]. The lncRNA-PCG pair was identified as significantly co-expressed in FANTOM5 samples if it passed the threshold used in [8], i.e., that their co-expression is greater than 75th percentile of the matched background correlation (*binom_p* < 0.05 compared to the background). To identify the eQTL that are co-localizing with GWAS SNP, eQTLs linking Wnt-regulated lncRNA and protein-coding genes were first mapped to SNP id using biomart in R. These SNPs were overlapped with trait-associated SNPs curated by FANTOM5 to select the SNPs associated with cancer by GWAS. In total, 271 eQTL SNPs were found to be associated with cancer by GWAS, linking 115 Wnt-regulated lncRNA-PCG pairs involving 49 Wnt-regulated lncRNAs (Additional file 5: Table S4).

### eQTL-linked lncRNA-PCG co-expression

The co-expression of eQTL-linked Wnt-regulated lncRNA-PCG pairs were examined in (1) FANTOM5, (2) TCGA Pancreatic Cancers, and (3) in response to Wnt inhibition in our system. Among the 1486 lncRNA-PCG pairs, the expression profiles of 1396 pairs are available in all three datasets (Additional file 6: Table S5). The lncRNA-PCG pairs’ co-expression coefficients and associated statistical significance in FANTOM5 were extracted from eQTL_linked_lncRNA_mRNA_pair [8]. Gene expression HTSeq-FPKM data of all the TCGA Pancreatic Cancers (PAAD) were downloaded from the UCSC Xena platform [39]. Only tumor samples were selected from the data, which yielded gene expression of 177 pancreatic cancer samples. The lncRNA-PCG pair co-expression in pancreatic cancer was calculated using the Spearman correlation *rho* on gene expression FPKM across the 177 samples. The associated *p* value was also calculated using cor.test function in R. The lncRNA-PCG pair co-expression in response to Wnt inhibition was calculated using the Spearman correlation *rho* on gene expression TPM across time points. The associated *p* value was also calculated using cor.test function in R.

### Time-series clustering

Time-series clustering on 10,554 Wnt-regulated genes, including 1503 Wnt-regulated lncRNAs and 9051 Wnt-regulated PCGs, was performed using GPClust [41] as previously described [26]. The clustering approach identifies both primary/early and secondary/late transcriptome responses to Wnt inhibition. It is based on two components—identification of a mean/covariance function for a specific cluster (using Gaussian processes) and determining the optimal number of clusters that can best model the data (using Dirichlet distribution) [41, 42]. Gene expression values were converted to *z*-scores, and time points were square root transformed. Genes were clustered with GPClust [41] using the Matern32 kernel with a length scale of 6 and a concentration (alpha) parameter of 0.001, 0.01, 0.1, 1, and 10. Genes were assigned to a cluster based on the highest probability of being a member of that cluster. Clustering was performed 10 times for a specified set of parameters, with the best clustering result taken as the one with the lowest distance to the other clustering results, i.e., the most representative (Additional file 7: Table S6).

### Functional enrichment analysis

Gene Ontology (GO) enrichment analysis was performed by g:Profiler [43] using all the Wnt-regulated protein-coding genes as background. Significantly enriched GO terms were selected with FDR < 5% (Additional file 7: Table S6).

### Enrichment analysis for dysregulated genes from different cancers

Genes significantly differentially expressed (adjusted *P* value < 0.05) between tumor-normal pairs were defined as dysregulated genes. To test whether the clusters were enriched for dysregulated genes in each cancer type, genes from each of the 63 clusters were tested for overlap against dysregulated genes from each cancer separately by carrying out a Fisher’s exact test. The gene background used for the test was Wnt-regulated genes that were expressed in the specific cancer. Upregulated genes and downregulated genes were examined separately for enrichment. The Fisher exact test for overrepresentation was performed using the fisher.test in R. Nominal *p* values were adjusted for multiple testing using the Benjamini-Hochberg method. Clusters significantly enriched for dysregulated genes were selected with FDR < 5%. The significance of the enrichment was then combined for each cluster and its enriched cancer type.

### CRISPRi sgRNA library design

CRISPRi single guide RNA (sgRNA) library was designed to target the transcription start site (TSS) of each of the Wnt-regulated lncRNAs. A total of 1503 Wnt-regulated lncRNAs were selected for the CRISPRi screen, which contained 3151 transcripts including different isoforms. To avoid redundancy of different TSSs located in close proximity, if TSSs of transcripts belonging to the same gene were within 100 bp, they were grouped together. A total set of 2337 TSSs were obtained for Wnt-regulated lncRNAs, which were then converted to hg19 with the liftover function in R. These TSSs were furthered refined with FANTOM based TSS annotation and 5 sgRNAs were designed to target each of the TSS using hCRISPRi-v2.1 algorithm [44]. Since some TSSs could not be uniquely targeted, in total 8560 sgRNAs were designed to target 1486 Wnt-regulated lncRNAs. The sgRNAs were then divided into 3 sub-libraries. Protein-coding genes whose TSSs were within 10 kb of Wnt-regulated lncRNAs were selected. sgRNAs targeting these protein-coding genes were extracted from the hCRISPRiv2 library [44] to constitute a 4th sub-library. For each sub-library, we also included 55 sgRNAs targeting 11 genes (*PCNA*, POLR2A, *PSMA7*, *RPS27*, *SF3A3*, *CTNNB1*, *FZD5*, *APC*, *AXIN1*, *CSNK1A1*, *PORCN*) involved in cell survival and Wnt signaling as positive controls and 50 non-targeting controls (Additional file 8: Table S7). The sgRNAs libraries were synthesized by CustomArray (Bothell, WA, USA).

### sgRNA cloning and lentiviral packaging

The sgRNA libraries were cloned into pCRISPRia-v2 sgRNA expression vector [44] by Gibson assembly (NEB). They were then amplified using electroporation in Endura electrocompetent cells (Lucigen), to achieve at least 250 colonies per sgRNA in the library. For CRISPRi knockdown validation, sgRNAs targeting *VPS9D1-AS1* and *XLOC_017401* were selected with protospacer sequences: sg*VPS9D1-AS1* (GAGCCAAGTCGCCCTGACCC), sg*XLOC_017401 (*GTCTGCTGCCAAGGAATCGG*).* The sgRNAs were cloned into pCRISPRia-v2 sgRNA expression vector as previously described [45]. The original pCRISPRia-v2 sgRNA expression vector contains protospacer sequence targeting GFP. For CRISPRi screen validation, top 2 performing sgRNAs targeting *LINC00263* and *SCD* were selected with protospacer sequences: sg*LINC00263_1* (GACCTCAGTCTGCCCTACCC), sg*LINC00263_2* (GGGTAGGGCAGACTGAGGTC), sg*SCD*_1 (GCTTGGCAGCGGATAAAAGG), sg*SCD*_2 (GCACATTCCCAACTCACGGA). The sgRNAs were cloned into doxycycline-inducible lentiviral sgRNA expression vector FgH1tUTG as previously described [46]. The sgRNA plasmid was packaged into lentiviral particles with psPAX2 and pMD2.G packaging plasmids. The virus supernatant was harvested 48 and 72 h after transfection, filtered through 0.45-μm filter, and stored at − 80 °C.

### Cell lines

The HPAF-II cell line was obtained from the Duke Cell Culture Facility. An HPAF-II stable cell line expressing dCas9-KRAB was generated by lentiviral transduction with pMH0001 plasmid (UCOE-SFFV-dCas9-BFP-KRAB) [47] and sorting for the top 20–30% BFP expressing cells. HPAF-II-dCas9-KRAB cells that stably express GFP were generated by lentiviral transduction with FUGW plasmid (Addgene plasmid # 14883) and sorting for the top 20–30% GFP expressing cells. For individual sgRNA knockdown using lentiviral sgRNA expression vector pCRISPRia-v2, cells were infected with sgRNA lentiviruses for 48 h, followed by 3 days of puromycin selection (2 μg/mL) and 1-day recovery to generate stable cell lines. For individual sgRNA knockdown using doxycycline-inducible lentiviral sgRNA expression vector FgH1tUTG, virally transduced cell lines were sorted for the GFP-positive cell population as previously described [46]. All cell lines were cultured in Eagle’s Minimum Essential Medium (EMEM) supplemented with 10% FBS, 1 mM sodium pyruvate, 2 mM l-glutamine, and 10% penicillin/streptomycin, maintained in 5% CO_2_. Cells were regularly tested for mycoplasma.

### CRISPRi screens

The HPAF-II-dCas9-KRAB stable cell line was infected with sgRNA lentiviral libraries at a multiplicity of infection (MOI) < 0.3 with 8 μg/ml polybrene. The infected cells were selected with 2 μg/ml puromycin for 3 days (T0 population). 3 × 10^6^ cells from the T0 population were harvested and stored as a cell pellet at − 20 °C for sequencing. For the in vitro screen, cells from T0 population were passaged with a seeding density of 3 × 10^6^ cells at each passage to allow for 1000 times coverage of each sgRNA and cultured for 2 weeks. 3 × 10^6^ cells at the end of the in vitro screen were harvested and stored as a cell pellet at − 20 °C for sequencing. The in vitro screen was performed in duplicates for each sub-library.

For the in vivo screen, NOD-scid gamma (NSG) mice were purchased from InVivos, Singapore, or Jackson Laboratories, Bar Harbor, Maine. Animal studies were approved by the Duke-NUS Institutional Animal Care and Use Committee. Mice were housed in standard cages and were allowed access ad libitum to food and water. Cells from the T0 population were mixed with ice cold 50% Matrigel (BD Biosciences) in PBS and injected subcutaneously into the flanks of NSG mice. 10^7^ cells resuspended in 200 μl Matrigel/PBS were injected per flank to allow for library coverage of 3000 cells/sgRNA at the time of implantation. A group of 3 mice were injected per sub-library. Mice were sacrificed 3 weeks after injection. At sacrifice, tumors were resected and snap frozen in liquid nitrogen at − 80 °C.

Genomic DNA from the frozen cell pellets and homogenized tumors was extracted with in-house high salt precipitation protocol. The sgRNA region integrated into the HPAF-II-dCas9-KRAB stable cell from the genomic DNA was amplified by PCR. A second round of PCR was performed to append Illumina sequencing adaptors and barcodes for each sample. PCR products were purified and quantified with a Bioanalyzer and sequenced on the Illumina MiSeq platform.

### CRISPRi screens’ analysis

Reads from sequenced screening sgRNA libraries were demultiplexed based on sample barcodes with FASTX-Toolkit. The reads were then counted against individual sub-libraries using MAGeCK count function [48] with non-targeting control sgRNA for normalization (Additional file 9: Table S8). sgRNA counts were used for quality control using PCA and clustering analysis with DESeq2 [37] to exclude outlier samples. Robust Rank Aggregation analysis (RRA) was performed with MAGeCK [48] test function to detect sgRNAs significantly depleted or enriched from the screens. Gene-level significance was calculated based on the performance of all its sgRNAs compared to non-targeting controls, as previously shown [48]. Each gene was also scored based on the fold change of its second best performing sgRNA [48]. We classified genes as hits if their associated FDR < 10% (Additional file 10: Table S9).

### Individual sgRNA CRISPRi knockdown

For individual sgRNA knockdown using doxycycline-inducible lentiviral sgRNA expression vector FgH1tUTG, 1 μg/ml doxycycline final concentration (dox) (from a stock of 10 mg/ml dissolved in DMSO) was used to induce sgRNA expression from the system, while DMSO was used as the control. After 48 h induction, total RNA was isolated from the CRISPRi knockdown cells. RT-qPCR was performed to assess the knockdown efficiency for *LINC00263 and SCD with HPRT* gene as an internal control*.* RT-qPCR primers were as follows: *LINC00263_*Forward (AAAGATTGGGCAGTCACTGG), *LINC00263_*Reverse (TGGGTCTTCAGCACCAAATG), *SCD_*Forward (TTCCTACCTGCAAGTTCTACACC), *SCD_*Reverse (CCGAGCTTTGTAAGAGCGGT)*.* The effect of CRISPRi knockdown on cell growth was assessed with internally controlled, relative growth assays. Cells were seeded in duplicates and treated with either 1 μg/ml dox or DMSO. Cells were counted every 3–4 days after the initial dox treatment. For individual sgRNA knockdown using lentiviral sgRNA expression vector pCRISPRia-v2, after generating stable cell line with sgRNA expression, total RNA was isolated from the CRISPRi knockdown cells. RT-qPCR was performed to assess the knockdown efficiency for *VPS9D1-AS1* and *XLOC_017401 with ACTB* gene as an internal control*.* RT-qPCR primers were as follows: *VPS9D1-AS1_*Forward (GTGTCTGGACACCAGAGGAGT), *VPS9D1-AS1_*Reverse (GGGGCAGAGTCACAAAGC)*, XLOC_017401_*Forward (GCCAGGCACACAGCAGTTTCTCA), *XLOC_017401_*Reverse (CCTAAGGAAGGTCCCGCCCCA)*.* For CRISPRi validation using sgRNA to target *GFP*, the GFP levels were measured from the parental HPAF-II-dCas9-KRAB cell line, HPAF-II-dCas9-KRAB-GFP cell line, HPAF-II-dCas9-KRAB-GFP stable cell line that express sg*GFP*, and HPAF-II-dCas9-KRAB-GFP stable cell line that express non-targeting control sgRNA, by flow cytometry.

## Results

### Discovery of Wnt-regulated lncRNAs

The HPAF-II pancreatic cancer cells contain a *RNF43* missense mutation that makes them addicted to Wnt signaling. As previously reported, mice with established orthotopic HPAF-II xenografts were treated with the PORCN inhibitor ETC-159 for 7 days. Tumors were harvested for transcriptomic analysis at indicated time points (0, 3, 8, 16, 32, 56, and 168 h) after starting ETC-159 treatment. The data were previously analyzed with a focus on protein-coding genes and splice variants [26, 49]. To demonstrate the specificity of ETC-159, we investigated the expression of 3 well-established Wnt target genes (*AXIN2*, *NKD1*, *LGR5*) in response to ETC-159 in WT HPAF-II cells and HPAF-II cells that express stabilized β-catenin (Additional file 1: Fig. S1A). All three Wnt target genes were significantly downregulated with ETC-159 treatment in HPAF-II cells; this inhibition was rescued by the expression of stabilized β-catenin (Additional file 1: Fig. S1A), demonstrating that the effect of ETC-159 is through inhibiting the Wnt/β-catenin pathway.

**Fig. 1:**
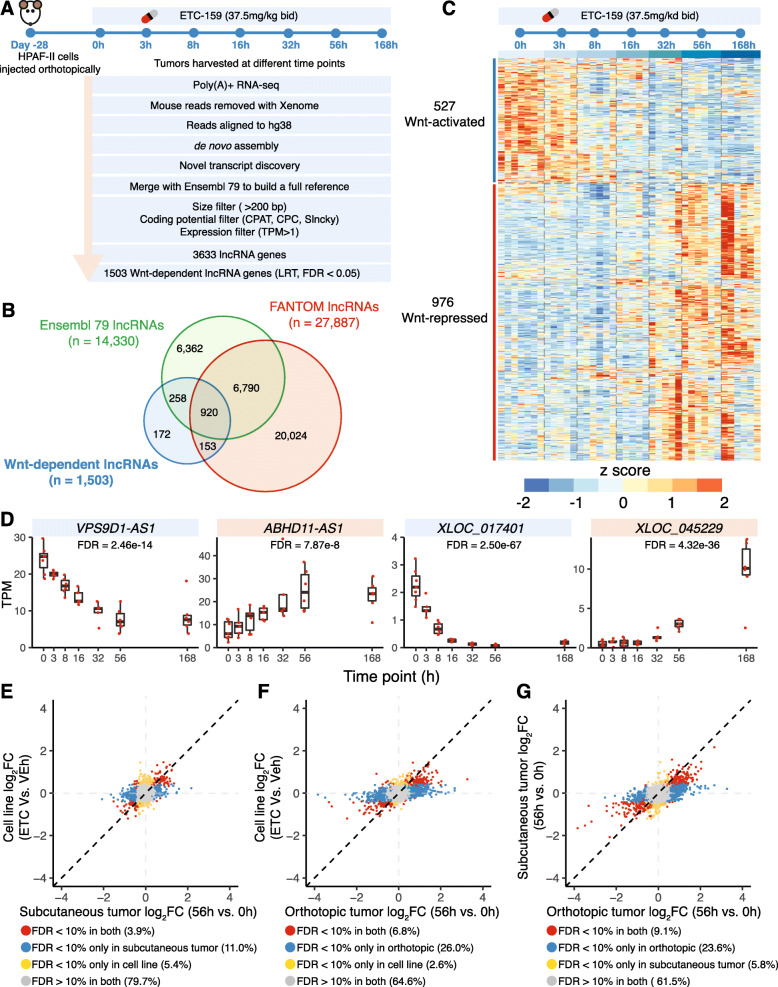
Identification of Wnt-regulated lncRNAs from orthotopic *RNF43*-mutant pancreatic cancer model. **a** Computational pipeline to identify 1503 Wnt-regulated lncRNAs from orthotopic *RNF43*-mutant pancreatic cancer. **b** Comparison of Wnt-regulated lncRNAs with Ensembl build 79 and FANTOM5 lncRNA annotations. **c** Expression profiles of 1503 Wnt-regulated lncRNAs across time points after Wnt inhibition. **d** Gene expression of selected Wnt-regulated lncRNAs, including annotated lncRNAs (*VPS9D1-AS1* and *ABHD11-AS1*) and novel lncRNAs (*XLOC_017401* and *XLOC_045229*). TPM, transcripts per million. **e–g** Fold change of lncRNAs after Wnt inhibition compared across models. More lncRNAs respond to Wnt inhibition in the HPAF-II subcutaneous (**e**) and orthotopic models (**f**) than in HPAF-II cells cultured in vitro. FC, fold change. **g** More lncRNAs respond to Wnt inhibition in HPAF-II orthotopic model than in the subcutaneous model

To comprehensively identify Wnt-regulated lncRNAs in pancreatic cancer in vivo, we reanalyzed this time-course transcriptomic dataset (Fig. 1a). We first used de novo assembly to comprehensively identify all the putative transcripts in this Wnt-addicted pancreatic xenograft model. These transcripts were then compared with the Ensembl build 79 transcriptome to identify putative novel lncRNAs. The putative novel lncRNAs were filtered based on their length (> 200 bp), and we eliminated those with coding potential called by any of three computational tools: CPAT [34], CPC [35], and Slncky [36] (see the “Methods” section for details). The novel lncRNAs were combined with previously annotated lncRNAs from Ensembl build 79 to establish a comprehensive list of lncRNAs present in our RNA-seq dataset. We next selected all the lncRNA genes with TPM > 1. Using these stringent criteria, we identified a set of 3633 lncRNAs in an orthotopic *RNF43*-mutant pancreatic cancer model (Fig. 1a). Among these 3633 lncRNAs, we found that the expression of 1503 lncRNAs changed over time upon Wnt inhibition (false discovery rate (FDR) < 5%); therefore, we refer to these lncRNAs as “Wnt-regulated lncRNAs” (Additional file 2: Table S1). Among the 1503 Wnt-regulated lncRNAs, 325 lncRNAs were not annotated in Ensembl build 79. We further compared these novel lncRNAs with FANTOM5 lncRNA annotations [8] and found 172 lncRNAs that have not been previously annotated either in Ensembl or FANTOM5 (Fig. 1b).

We found that twice as many lncRNAs were upregulated (976 Wnt-repressed lncRNAs) than downregulated (527 Wnt-activated lncRNAs) following PORCN inhibitor treatment (Fig. 1c). Among them, 240 Wnt-repressed and 85 Wnt-activated lncRNAs are not annotated in Ensembl build 79. The 527 Wnt-activated lncRNAs responded as early as 3 h after the first dose of ETC-159, consistent with direct regulation by Wnt/β-catenin signaling. Conversely, the 976 Wnt-repressed lncRNAs responded more slowly to Wnt inhibition (Fig. 1c), which could be due to indirect Wnt regulation. For example, *VPS9D1-AS1*, a previously reported target of Wnt/MYC signaling [27], was downregulated rapidly after PORCN inhibitor treatment and the inhibition was sustained for 7 days. Similarly, a previously unannotated lncRNA *XLOC_017401* was also downregulated shortly after Wnt inhibition. In contrast, *XLOC_045229*, another previously unannotated lncRNA, was upregulated after ETC-159 treatment, but the effect was only observed after 32 h of treatment (Fig. 1d). As we showed for well-established Wnt target genes, we confirmed that the effect of ETC-159 on these Wnt-regulated lncRNAs is indeed through modulating Wnt/β-catenin signaling (Additional file 1: Fig. S1). Taken together, we identified 1503 lncRNAs whose expression is regulated either directly or indirectly by Wnt signaling in vivo in an *RNF43*-mutant pancreatic cancer.

Genes that are important in cancer pathogenesis can be regulated by multiple pathways. For example, the well-known proto-oncogene *MYC* can be activated by pathological Wnt signaling in Wnt-driven cancers and also by diverse additional pathways in other cancers [50]. Similarly, we postulated that if a specific Wnt-regulated lncRNA is important in cancer, the same lncRNA might also be dysregulated by other mechanisms in other cancer types. To test this, we analyzed gene expression data from TCGA [39], comparing tumors with their paired normal samples. We found that many Wnt-regulated lncRNAs were also dysregulated in different and Wnt-independent types of cancers (Additional file 1: Fig. S2A). For example, we identified that between 246 and 435 Wnt-regulated lncRNAs were significantly upregulated in various cancer types compared to their paired normal samples (Additional file 1: Fig. S2A). We found only 68 significantly upregulated and 10 significantly downregulated Wnt-regulated lncRNAs in pancreatic cancer (Additional file 1: Fig. S1A, Additional file 3: Table S2); this low number is because only 4 pairs of pancreatic cancer-normal samples are present in the TCGA dataset, limiting the statistical power (Additional file 1: Fig. S2B). We also found 248 Wnt-regulated lncRNAs exclusively upregulated or downregulated across different cancer types (Additional file 2: Table S1). For example, *VPS9D1-AS1*, a known Wnt/MYC target, was both Wnt-activated in our study and also upregulated in 11 different types of cancers (Additional file 1: Fig. S2C), consistent with its established role as a lncRNA with oncogenic function [27]. Together, these analyses suggest that a subset of Wnt-regulated lncRNAs can act as mediators of oncogenic processes in both Wnt-dependent and Wnt-independent cancers, and in multiple cancer types beyond Wnt-addicted pancreatic cancer.

### LncRNAs respond to Wnt inhibition more robustly in vivo, especially in orthotopic xenograft model

Tumor microenvironment is important for tumor pathogenesis [51–53]. To examine how the response of lncRNAs to Wnt inhibition is affected by the stromal microenvironment, we compared the effect of ETC-159 on lncRNAs expression in HPAF-II orthotopic or subcutaneous xenografts (in vivo) and in cultured cells (in vitro). Nearly twice as many lncRNAs responded to the PORCN inhibitor treatment in the subcutaneous xenograft (541/3633) compared to those that responded in vitro (341/3633) (Fig. 1e). A further increase in the number of lncRNAs responding to Wnt inhibition was observed in the orthotopic xenografts (1191/3633) (Fig. 1f). This is consistent with our previous observation that Wnt-regulated gene expression changes are more robust in vivo [26]. Interestingly, between the two in vivo models, many more lncRNAs responded to Wnt inhibition in the orthotopic than subcutaneous xenograft (Fig. 1g). This is consistent with our previous observation that the overall changes in gene expression following Wnt inhibition were most marked in the orthotopic model [26]. Taken together, this indicates that in vivo models can substantially enhance the discovery of Wnt-regulated genes, including lncRNAs.

### A subset of Wnt-regulated lncRNAs are co-expressed with their nearest protein-coding gene in the same TAD

Most of the Wnt-regulated lncRNAs identified here have not previously been described or functionally characterized. Since lncRNAs can be important regulators of nearby genes [54–56], we set out to explore their potential *cis* functions. If a lncRNA and its nearby protein-coding gene (PCG) are positively co-expressed after Wnt inhibition, it suggests that the lncRNA and its PCG neighbor could be functionally linked. To test this, we analyzed the expression changes of lncRNAs and PCGs in response to PORCN inhibitor treatment. We found that on average, Wnt-regulated lncRNAs exhibited stronger co-expression with their nearest PCG after Wnt inhibition compared to their co-expression with all PCGs (Additional file 1: Fig. S3A). This stronger co-expression can be partially explained by the fact that some of the Wnt-regulated lncRNA–nearest PCG pairs are within the same topological associated domain (TAD) (Additional file 1: Fig. S3B), where they may functionally interact with each other more frequently, as previously suggested [57]. Interestingly, for these Wnt-regulated lncRNA–nearest PCG pairs encoded within the same TAD, the PCGs were significantly enriched for Gene Ontology (GO) biological processes such as organ development and cell fate specification (Additional file 1: Fig. S3C). This suggests that these highly co-expressed Wnt-regulated lncRNAs that are proximal to PCGs and co-localized within the same TAD are likely to be involved in the same cellular processes.

### Influence of Wnt inhibition on the co-expression relationship of lncRNAs and eQTL-linked protein-coding genes

**Fig. 2:**
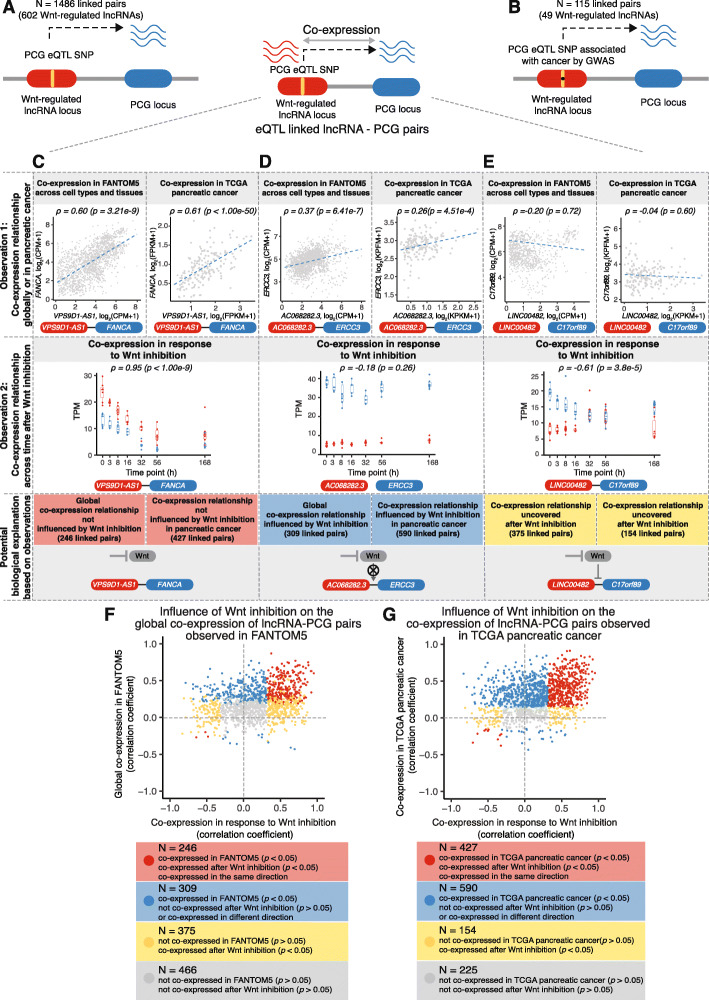
Influence of Wnt inhibition on the co-expression relationship of lncRNAs and their eQTL-linked protein-coding genes. **a** Wnt-regulated lncRNAs are linked to their nearby protein-coding genes (PCGs) if the eQTL SNP of a PCG overlaps with a lncRNA locus, as annotated by FANTOM5 consortium [8]. **b** 115 Wnt-regulated lncRNA-PCG pairs are linked by eQTL SNPs that also colocalize with cancer GWAS loci. **c** Wnt-regulated lncRNA *VPS9D1-AS1* and its eQTL-linked PCG *FANCA* are co-expressed in both FANTOM5 and TCGA pancreatic cancer. They are also co-expressed after Wnt inhibition, suggesting their co-expression is not directly influenced by Wnt signaling inhibition. CPM, counts per million. FPKM, fragments per kilobase of transcript per million. **d** Co-expression of Wnt-regulated lncRNA *AC068282.3* and its eQTL-linked PCG *ERCC3* is observed in both FANTOM5 and TCGA pancreatic cancer and is influenced by Wnt signaling, as they are no longer co-expressed after Wnt inhibition in our system. **e** Wnt-regulated lncRNA *LINC00482* and its eQTL-linked PCG *C17orf89* are not co-expressed in either FANTOM5 or TCGA pancreatic cancer but become co-expressed in response to Wnt inhibition. **f**, **g** Influence of Wnt inhibition on the co-expression relationship of Wnt-regulated lncRNAs and their eQTL-linked PCGs observed in FANTOM5 (**f**) or TCGA pancreatic cancer (**g**). Red, co-expression of lncRNA-PCG pairs observed in FANTOM5 (**f**) or TCGA pancreatic cancer (**g**) are not directly influenced by Wnt inhibition; blue, co-expression of lncRNA-PCG pairs observed in FANTOM5 (**f**) or TCGA pancreatic cancer (**g**) are influenced by Wnt inhibition; yellow, co-expression of lncRNA-PCG pairs are uncovered after Wnt inhibition; gray, lncRNA-PCG pairs are neither co-expressed in response to Wnt inhibition nor in FANTOM5 (**f**) or TCGA pancreatic cancer (**g**)

Expression quantitative trait loci (eQTLs) analysis, which links DNA sequence variation with changes in gene expression, has been a powerful approach for understanding the functional effects of common SNPs [58]. The underlying regulatory mechanisms of the eQTL SNPs on gene expression depend on the genomic functional element perturbed by the genetic variant. For example, an eQTL SNP within a lncRNA might modify its interaction with transcription factors or epigenetic modifiers, thereby altering the expression of nearby PCGs [59]. SNPs within lncRNA loci that are associated with the mRNA abundance of nearby genes (< 1 Mbp apart), i.e., *cis*-acting regulation, have been systematically annotated by the FANTOM5 consortium to establish lncRNA-mRNA pairs linked by these eQTL SNPs [8]. This lncRNA-mRNA interaction mediated by an eQTL suggests that these lncRNAs loci might potentially regulate the expression of nearby mRNAs. The FANTOM5 dataset contains genome-wide transcriptome profiles of 1829 samples from more than 173 human primary cell types and 174 tissues across the human body, 276 cancer cell lines, and 19 time courses of cellular treatment. If the eQTL-linked lncRNA-mRNA are co-expressed in FANTOM5 samples, it further suggests a functional association between the lncRNA and its eQTL-linked mRNA. Here, to identify Wnt-regulated lncRNAs with potential regulatory effects on nearby PCG mRNAs, we overlapped 1503 Wnt-regulated lncRNAs with all of the lncRNA-mRNA pairs annotated by the FANTOM5 consortium. We found 1486 lncRNA PCG mRNA (lncRNA-PCG) pairs linked by eQTL SNPs involving 602 Wnt-regulated lncRNAs. (Some of the lncRNAs were linked to multiple PCGs (Fig. 2a and Additional file 4: Table S3).) Among them, 587 lncRNA-PCG pairs were also significantly co-expressed (*p* < 0.05) in FANTOM5 samples. This global co-expression relationship across FANTOM5 samples suggests that the Wnt-regulated lncRNA and its eQTL-linked PCG could be functionally linked broadly across cell types and tissues.

We then investigated the diseases associated with the Wnt-regulated lncRNA-PCG pairs linked by eQTLs, with a focus on cancer. eQTLs that co-localize with disease risk loci identified by genome-wide association studies (GWAS) are candidates for the regulation of complex traits and diseases, including SNPs associated with cancer susceptibility by GWAS [60]. We examined the eQTL SNPs overlapping with the Wnt-regulated lncRNAs loci and matched these SNPs with those curated by FANTOM5 for 56 cancer GWAS traits. Among the 1486 eQTL-linked Wnt-regulated lncRNA-PCG pairs, a subset of 115 pairs involving 49 Wnt-regulated lncRNAs were linked by eQTL SNPs that colocalize with cancer GWAS loci (Fig. 2b, Additional file 5: Table S4). For example, *AC068282.3* was linked to *ERCC3* through 4 distinct *ERCC3* eQTL SNPs that were also associated with leukemia by GWAS (Additional file 5: Table S4). In addition, *AC068282.3* showed global co-expression with *ERCC3* in FANTOM5 across cell types and tissues (*p* = 6.41e−7) (Fig. 2d). This might suggest that *AC068282.3* is involved in susceptibility to leukemia through its regulation of *ERCC3*. Integrating eQTL-linked Wnt-regulated lncRNA-PCG pairs with cancer GWAS data suggests that 3% (49/1503) of the Wnt-regulated lncRNAs may confer cancer susceptibility through their *cis*-regulation of eQTL-linked PCGs [59, 61].

Our time-series data on gene expression change in response to Wnt inhibition is unique in that only Wnt signaling is perturbed in this system. This allowed us to examine how the lncRNA-PCG pair co-expression is affected by Wnt inhibition in a temporal manner. We further examined if Wnt signaling inhibition influenced the co-expression relationship of Wnt-regulated lncRNAs and their eQTL-linked PCGs. To do this, we compared their co-expression detected in response to Wnt inhibition to their global co-expression observed in the FANTOM5 dataset (1829 samples) across cell types and tissues or their co-expression observed in TCGA pancreatic cancer (177 samples). First, we found 246 lncRNA-PCG pairs that were significantly co-expressed in both FANTOM5 and our dataset, irrespective of Wnt signaling status (Fig. 2f, Additional file 6: Table S5). We found more (427) lncRNA-PCG pairs that were significantly co-expressed in TCGA pancreatic cancer and the co-expression remained after Wnt inhibition (Fig. 2g, Additional file 6: Table S5). One illustrative example of this consistent co-expression pattern is *VPS9D1-AS1* (lncRNA) and *FANCA* (eQTL-linked PCG) in Fig. 2c. Here, the lncRNA-PCG co-expression was significant (*p* < 0.05) and had the same direction, i.e., positive in response to Wnt inhibition in our system and positive in FANTOM5 samples and TCGA pancreatic cancer samples. In this set of lncRNA-PCG pairs, their co-expression relationships were not directly influenced by Wnt signaling inhibition. Second, there were 309 and 590 lncRNA-PCG pairs significantly co-expressed in the FANTOM5 dataset and TCGA pancreatic cancer, which were either not significantly co-expressed or co-expressed in the opposite direction after Wnt inhibition (Fig. 2f, g, Additional file 6: Table S5). For example, *AC068282.3* and *ERCC3* were significantly co-expressed in both FANTOM5 samples and in TCGA pancreatic cancer (*p* < 0.05), but in response to Wnt inhibition, they were no longer co-expressed (*p* = 0.26) (Fig. 2d). This suggests that the co-expression relationship observed either globally or in TCGA pancreatic cancer could be influenced by Wnt signaling inhibition. Finally, a third group of Wnt-regulated lncRNA-PCG pairs (Fig. 2f and g, Additional file 6: Table S5), although linked by eQTL SNPs, were not significantly co-expressed across FANTOM5 samples (375 pairs) or TCGA pancreatic cancer (154 pairs). However, they became significantly co-expressed in a temporal manner responding to Wnt inhibition. For example, *LINC00482* and *C17orf89* were not correlated in either FANTOM5 or TCGA pancreatic cancer, but they became co-expressed in response to Wnt inhibition (Fig. 2e). Thus, the co-expression relationship of these lncRNAs and PCGs could be uncovered after Wnt inhibition. Taken together, these analyses suggest that Wnt signaling inhibition could influence the co-expression relationship of Wnt-regulated lncRNAs and their eQTL-linked PCGs. Therefore, Wnt signaling is important for both the regulation and the potential cis function of a subset of Wnt-regulated lncRNAs.

### Wnt-regulated lncRNAs and protein-coding genes form gene networks that are dysregulated in cancers

Beside *cis* regulatory functions, lncRNAs can also participate in gene networks that regulate diverse biological processes [62, 63]. Most of the Wnt-regulated lncRNAs have not previously been functionally characterized. By associating these lncRNAs with known protein-coding genes from the same gene network, we can infer their potential biological functions. To investigate which gene networks the various Wnt-regulated lncRNAs may be involved in, we performed time-series clustering of the differentially expressed Wnt-regulated lncRNAs and PCGs. This analysis closely paralleled a similar time-series clustering of PCGs that we reported previously, which allowed us to differentiate between primary/early and secondary/late transcriptional responses to Wnt inhibition [26]. The lncRNAs and PCGs fell into 63 distinct clusters based on their pattern of expression change following Wnt inhibition (Additional file 1: Fig. S4A, Additional file 7: Table S6). The similar and coherent dynamic response of each cluster to Wnt inhibition suggests the presence of a common regulatory process within each cluster [64].

**Fig. 3:**
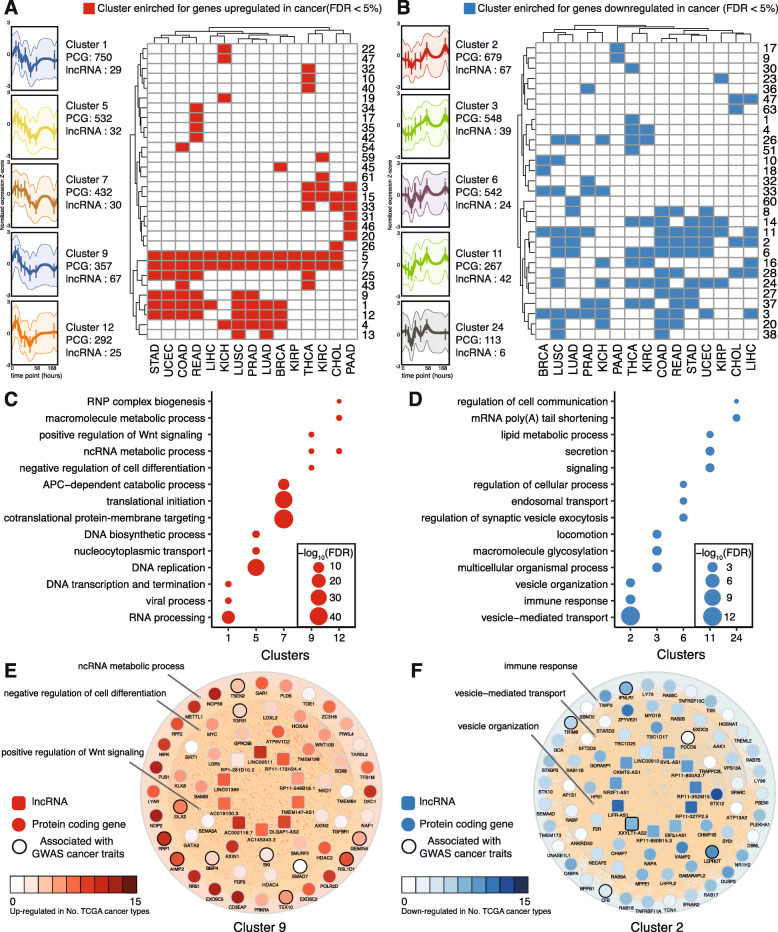
Wnt-regulated lncRNA and protein-coding genes form gene networks that are dysregulated in different cancer types. **a** Clusters enriched for genes upregulated in different cancer types. The top 5 clusters, clusters 1, 5, 7, 9, and 12, are enriched with the most number of cancers for genes upregulated. Normalized gene expression of these 5 clusters with number of PCGs and lncRNAs from each cluster are shown (left). **b** Clusters enriched for genes downregulated in different cancer types. The top 5 clusters, clusters 2, 3, 6, 11, and 24, are enriched with the most number of cancers for genes downregulated. Normalized gene expression of these 5 clusters with number of PCGs and lncRNAs from each cluster are shown (left). **c**, **d** GO Biological Processes enrichments (FDR < 5%) of the top 5 clusters enriched for genes upregulated (**c**) or downregulated (**d**) in different cancer types. The top 3 significantly enriched GO terms for each cluster are shown. **e** Wnt-regulated lncRNAs are part of gene networks that are upregulated in different cancers. PCGs from cluster 9 are enriched for ncRNA metabolic processes, negative regulation of cell differentiation, and positive regulation of Wnt signaling. Wnt-regulated lncRNAs from cluster 9 are shown in the inner circle. **f** Wnt-regulated lncRNAs are part of gene networks that are downregulated in different cancers. PCGs from cluster 2 are enriched for immune response, vesicle-mediated transport, and vesicle organization. Wnt-regulated lncRNAs from cluster 2 are shown in the inner circle

As many Wnt-regulated lncRNAs and PCGs were also dysregulated in different types of cancers as determined by differential expression between tumors and their paired normal samples in the TCGA dataset [39] (Additional file 1: Fig. S2A), we tested if the lncRNA-PCGs’ clusters were enriched for dysregulated genes in different cancer types. This helped us to prioritize the clusters that are more relevant to cancer. We also investigated the biological processes that are enriched in these clusters and then focused on the clusters that are enriched for processes related to Wnt signaling inhibition. We found that 48 out of the 63 clusters are enriched (FDR < 5%) for genes dysregulated in at least one type of cancer (Additional file 1: Fig. S4B). In addition, a number of clusters (clusters 1, 5, 7, 9, 12 and clusters 2, 3, 6, 11, 24) (Fig. 3a, b) are enriched for genes up- or downregulated in the majority of cancer types, suggesting that these gene networks play a broad role in the pathogenesis of cancer. For example, cluster 9 contained 67 Wnt-activated lncRNAs and 357 PCGs, including well-established Wnt target genes (e.g., *NKD1*, *AXIN2*, *LGR5*, *MYC*, *BMP4*, *FGF9*) (Fig. 3e, Additional file 7: Table S6). This cluster was enriched for genes upregulated in 6 cancer types and genes downregulated in pancreatic cancer (Fig. 3a, b). It was significantly enriched for ncRNA metabolic process, Wnt signaling, and cell differentiation (Fig. 3c, e). Many of the genes associated with ncRNA metabolic process (e.g. *NOP56*, *METTL1*, *RRP1*, *AIMP2*, *EXOSC5*) were also overexpressed in multiple cancers (Fig. 3e). With a few notable exceptions such as lncRNA *LINC00511* [65], most of the lncRNAs in this cluster do not have established biological functions. One the other hand, cluster 2 contained mainly Wnt-repressed genes, the majority of which were downregulated in eight cancer types (Fig. 3b, Additional file 7: Table S6). The PCGs from this cluster were enriched for processes related to vesicle organization, vesicle transport, and immune response (Fig. 3d, f). This last finding is consistent with recent studies demonstrating that Wnt signaling prevents anti-tumor immunity and suppresses immune surveillance [66, 67]. Although most of the lncRNAs from cluster 2 have not been characterized before, *LINC00910* was previously identified as a lncRNA highly connected to other gene promoter regions and was proposed to be involved in lymphocyte activation [68]. Taken together, this lncRNA-PCG network analysis suggests specific Wnt-regulated lncRNAs in gene networks are involved in distinct biological processes that contribute to the pathogenesis of cancers.

### CRISPRi screens identify Wnt-regulated lncRNAs that modify HPAF-II cell growth in a context-dependent manner

Our analysis identified multiple Wnt-regulated lncRNAs, a subset of which might be important in cancer progression. To specifically identify the lncRNAs that play functional roles in the pathogenesis of *RNF43*-mutant pancreatic cancer in vivo, we performed CRISPRi screens. This approach utilizes dCas9-KRAB, where a catalytically inactive Cas9 is fused to a Krüppel-associated box (KRAB) transcriptional repressor domain [69]. dCas9-KRAB is recruited to the transcription start site (TSS) of lncRNAs by single guide RNAs (sgRNAs) to repress the transcription of the lncRNA of interest. CRISPRi screens have been demonstrated to be an efficient and specific approach for genome-wide loss-of-function studies of lncRNAs [45], which cannot reliably be inactivated by indels introduced by the standard CRISPR-Cas9 system.

**Fig. 4:**
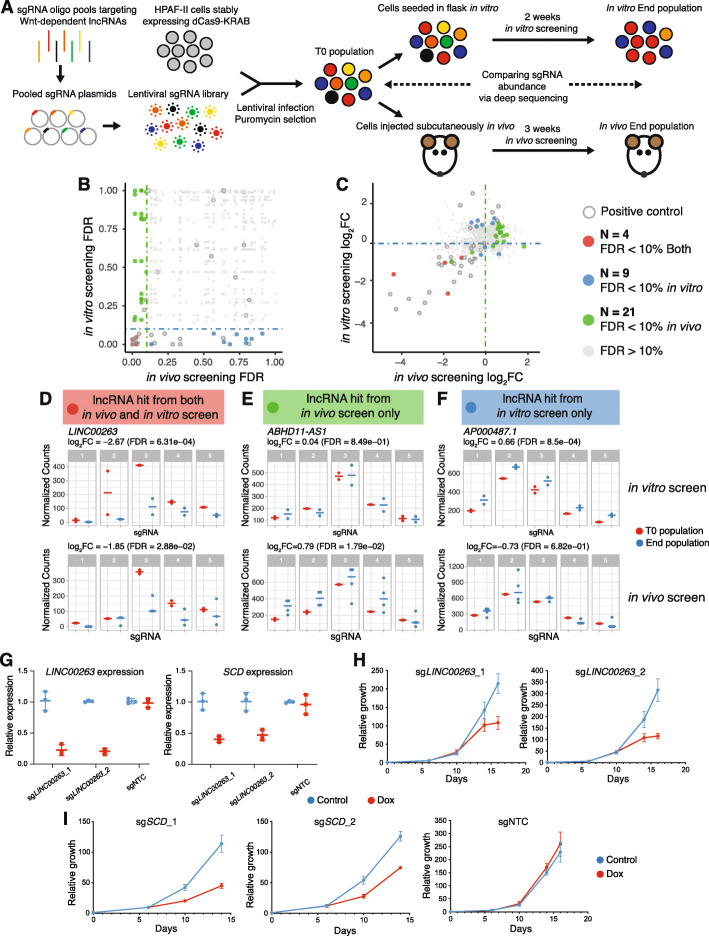
CRISPRi screens identify Wnt-regulated lncRNAs loci that modify cell growth in a context-dependent manner. **a** Schematic representation of CRISPRi screens conducted using xenograft tumors in vivo and in cultured cells in vitro to identify functional Wnt-regulated lncRNAs in *RNF43*-mutant pancreatic cancer. **b** Comparison of FDR from in vivo and in vitro screens. The dashed lines represent the threshold (FDR = 10%) for calling hits by gene-associated FDR. lncRNA hits are colored based on their FDR from both in vivo and in vitro screens. **c** Comparison of sgRNA fold change after in vivo and in vitro screens. Each gene is colored based on hits calling from B. **d** sgRNAs targeting *LINC00263* are significantly depleted from both in vivo and in vitro screens. **e** sgRNAs targeting *ABHD11-AS1* are significantly enriched only from the in vivo screen. **f** sgRNAs targeting *AP000487.1* are significantly enriched only from the in vitro screen. The normalized counts of 5 sgRNAs targeting the TSS of *LINC0026*3, *ABHD11-AS1*, and *AP000487.1* are shown before and after both screens in **d**–**f**. **g** sgRNAs targeting the TSS of *LINC00263* reduce the expression of *LINC00263 and SCD*. **h** sgRNAs targeting *LINC00263* reduce HPAF-II cell growth in vitro. Cell numbers were counted at days 6, 10, 14, and 16 after seeding and normalized to the seeding density. **i** sgRNAs targeting *SCD* reduce HPAF-II cell growth in vitro. sgNTC does not affect cell growth. Cell numbers were counted at days 6, 10, and 14 after seeding and normalized to the seeding density. NTC, non-targeting control

We chose to perform this CRISPRi screen in vivo because we have shown that both lncRNAs and PCGs respond to Wnt inhibition more robustly in vivo (Fig. 1e-g and [26]) and that in vivo screening identifies dependencies not seen in tissue culture [70]. To capture the difference of Wnt-regulated lncRNA functions in vivo and in vitro, the CRISPRi screen was conducted both using xenograft tumor in vivo as well as cultured cells in vitro (Fig. 4a).

We designed five sgRNAs to target the TSS of each of the 1503 Wnt-regulated lncRNAs [44]. We divided the sgRNAs into 3 lentiviral sub-libraries to allow for full representation of the sgRNAs throughout the in vivo screen, due to the limited number of cells that can be implanted and engrafted in each tumor. For each sub-library, we also included 55 sgRNAs targeting 11 genes involved in cell survival or Wnt signaling as positive controls, and 50 non-targeting controls (Additional file 8: Table S7). To assess the efficacy of this CRISPRi system for gene suppression, we first selected sgRNAs targeting two Wnt-regulated lncRNAs, including an annotated lncRNA *VPS9D1-AS1* and a novel lncRNA *XLOC_017401*. We found that the CRISPRi system suppressed *VPS9D1-AS1* expression by ~ 85% and *XLOC_017401* expression by ~ 70% (Additional file 1: Fig. S5A). We also generated HPAF-II-dCas9-KRAB cells that stably expressed GFP and confirmed in these cells that a specific sgRNA was able to knock down GFP expression (Additional file 1: Fig. S5B).

We transduced the HPAF-II cell line stably expressing dCas9-KRAB with the lentiviral sgRNA sub-libraries at a low multiplicity of infection (MOI < 0.3) to ensure that each cell was only infected by one virus with a single sgRNA. The transduced cells were selected with puromycin for 3 days (T0 population) and then maintained in culture for 2 weeks (the in vitro screen) with ≥ 3 × 10^6^ cells to allow for 1000-fold coverage of each sgRNA throughout the in vitro screen. Alternatively, the transduced cells were injected subcutaneously into immunocompromised mice. To get a good representation of each guide in the subcutaneous tumor, a total of 10^7^ cells were injected per mouse flank to allow for 3000-fold coverage of each sgRNA. The tumors were harvested after 3 weeks (the in vivo screen). Integrated lentiviruses encoding sgRNAs (i.e., barcodes) from the T0 population, the in vitro screen end population, and the in vivo screen end population were then recovered by PCR and quantified by next-Gen sequencing (see the “Methods” section for additional details, Additional file 9: Table S8).

We first assessed the technical quality of the CRISPRi screen. There was a high correlation of sgRNA frequencies between independent experimental replicates (Additional file 1: Fig. S6), suggesting the robustness of the screen. We used the MAGeCK algorithm [48] to analyze the in vitro and in vivo screens, using the non-targeting control sgRNAs for normalization. The statistical determination that a lncRNA gene regulated cancer proliferation was calculated based on the performance of all its sgRNAs compared to the non-targeting controls, as previously reported [48]. Each lncRNA gene was also scored based on the fold change of its second best performing sgRNA [48]. We classified a gene as a hit if its associated FDR was less than 10% (Fig. 4b). First, our screen was able to identify important positive controls as gene hits. For example, 4 out of 5 sgRNAs targeting *POLR2A* (RNA polymerase II subunit A) were depleted in both in vitro and in vivo screens, consistent with its essential role for cell growth (Additional file 1: Fig. S7). As expected for a Wnt-addicted cancer, all 5 sgRNAs targeting *CTNNB1* were also depleted in both in vitro and in vivo screens (Additional file 1: Fig. S7). Thus, the screen appears to function well both in vitro and in vivo.

We next compared the lncRNA hits from the in vivo and in vitro screens. We identified 4 Wnt-regulated lncRNA loci as hits in both screens, 21 lncRNA loci as hits only in the in vivo screen and 9 lncRNA loci as hits only in the in vitro screen (Fig. 4b and c, and Table 1). Since CRISPRi acts within a 1-kb window around the targeted TSS to repress gene expression [71], we also included in our sgRNA library guides designed to suppress the expression of the protein-coding genes that also had a TSS within 1 kb of the TSS of lncRNA hits. We found that for 6 lncRNA hits, protein-coding genes were nearby that could be suppressed by CRISPRi in the screen. However, CRISPRi suppression of these protein neighbors did not produce a phenotype in a separate screen library (Additional file 10: Table S9). This indicates that the lncRNA hits identified through CRISPRi screen are likely due to the functions of lncRNA loci themselves. Taken together, around 1% (13/1503) of the Wnt-regulated lncRNAs can modify cancer cell growth in the in vitro screen, which is consistent with previous genome-wide CRISPRi screens for functional lncRNAs in cell lines [45]. Notably, using the in vivo CRISPRi screen, we identified twice as many Wnt-regulated lncRNAs (25/1503) that had a functional effect on cancer cell growth.

**Table 1:** Wnt-regulated lncRNAs that affect HPAF-II cell growth in vivo and in vitro

Group	Ensembl Gene ID	Gene symbol	Gene biotype	log2FC^**a**^ (in vivo)	FDR^**b**^ (in vivo)	log2FC (in vitro)	FDR (in vitro)	Wnt-dependence	Nearest PCG	Correlation (nearest PCG)^**c**^	***p value*** of correlation	lncRNA-PCG Distance (bp)^**d**^	Upregulated No. cancers^**e**^	Downregulated No. cancers^**f**^
in vitro and in vivo	*ENSG00000276131*	*RP11-481J2.3*	antisense	− 4.42	**0.001**	− 1.76	**0.001**	Wnt-Activated	*GINS3*	0.67	3.65E−06	97,726	6	0
in vitro and in vivo	*ENSG00000188825*	*LINC00910*	lincRNA	− 1.99	**0.027**	− 1.22	**0.045**	Wnt-Repressed	*ARL4D*	0.39	1.40E−02	9759	1	4
in vitro and in vivo	*ENSG00000235823*	*LINC00263*	lincRNA	− 1.85	**0.029**	− 2.67	**0.001**	Wnt-Repressed	*SCD*	0.54	4.04E−04	26,490	6	1
in vitro and in vivo	*ENSG00000247844*	*CCAT1*	lincRNA	− 1.20	**0.001**	− 0.99	**0.033**	Wnt-Activated	*MYC*	0.80	3.42E−08	516,345	9	3
in vivo	*ENSG00000230177*	*RP5-1112D6.4*	antisense	− 0.69	**0.096**	− 0.75	0.991	Wnt-Repressed	*KIAA1919*	− 0.24	1.29E−01	18,583	10	0
in vivo	*ENSG00000230266*	*XXYLT1-AS2*	antisense	1.77	**0.098**	− 0.46	0.471	Wnt-Repressed	*XXYLT1*	− 0.13	4.24E−01	123,295	1	7
in vivo	*ENSG00000233895*	*RP1-122P22.2*	lincRNA	− 1.65	**0.015**	− 1.18	0.158	Wnt-Repressed	*RIN2*	0.63	2.36E−05	128,812	0	10
in vivo	*ENSG00000259146*	*RP1-261D10.2*	antisense	0.22	**0.063**	0.12	0.541	Wnt-Activated	*SIPA1L1*	0.45	3.79E−03	1364	2	5
in vivo	*ENSG00000259985*	*RP11-549B18.1*	antisense	0.60	**0.063**	0.22	0.329	Wnt-Activated	*B4GALT6*	0.81	2.04E−08	*180*	6	4
in vivo	*ENSG00000261662*	*RP5-1042I8.7*	sense_overlapping	0.30	**0.018**	− 0.45	1.000	Wnt-Repressed	*NOTCH2*	0.40	1.19E−02	159,012	1	9
in vivo	*ENSG00000233912*	*AC026202.3*	antisense	0.66	**0.063**	0.29	0.295	Wnt-Activated	*ARL8B*	− 0.23	1.52E−01	65,108	2	7
in vivo	*ENSG00000262903*	*RP11-235E17.6*	antisense	0.48	**0.063**	− 0.01	1.000	Wnt-Repressed	*CTNS*	− 0.12	4.61E−01	21,623	8	1
in vivo	*XLOC_052899*	*XLOC_052899*	novel_lncRNAs	0.66	**0.063**	0.43	0.813	Wnt-Repressed	*TMEM161B*	0.68	2.75E−06	393,258	NA	NA
in vivo	*ENSG00000234477*	*AC004231.2*	antisense	0.96	**0.018**	0.09	0.974	Wnt-Repressed	*KRT23*	0.74	2.93E−07	16,203	5	2
in vivo	*ENSG00000225969*	*ABHD11-AS1*	antisense	0.79	**0.018**	0.04	0.849	Wnt-Repressed	*ABHD11*	0.69	1.75E−06	3828	9	3
in vivo	*ENSG00000196421*	*LINC00176*	lincRNA	0.57	**0.063**	0.25	0.823	Wnt-Activated	*ZNF512B*	0.09	5.76E−01	14,413	10	0
in vivo	*ENSG00000272379*	*RP1-257A7.5*	lincRNA	0.75	**0.063**	0.49	0.295	Wnt-Repressed	*TBC1D7*	− 0.13	4.30E−01	38,092	1	1
in vivo	*XLOC_001141*	*XLOC_001141*	novel_lncRNAs	0.81	**0.063**	− 0.24	1.000	Wnt-Activated	*DEPDC1*	0.08	6.35E−01	30,939	NA	NA
in vivo	*XLOC_022655*	*XLOC_022655*	novel_lncRNAs	0.67	**0.084**	0.20	0.813	Wnt-Activated	*DIS3L*	0.77	1.09E−07	42,903	NA	NA
in vivo	*ENSG00000232536*	*RP11-74C1.4*	sense_intronic	0.63	**0.018**	0.18	0.541	Wnt-Repressed	*TUFT1*	0.40	1.08E−02	*210*	5	2
in vivo	*ENSG00000262468*	*LINC01569*	lincRNA	0.56	**0.063**	0.62	0.160	Wnt-Activated	*TFAP4*	0.74	3.28E−07	19,285	9	1
in vivo	*ENSG00000184224*	*C11orf72*	lincRNA	0.52	**0.063**	0.36	0.272	Wnt-Repressed	*NDUFV1*	0.11	5.10E−01	*145*	3	2
in vivo	*ENSG00000277692*	*RP11-358N2.2*	lincRNA	0.50	**0.063**	− 0.27	1.000	Wnt-Repressed	*ASXL1*	− 0.36	2.43E−02	3290	3	3
in vivo	*ENSG00000250413*	*RP11-448G15.1*	antisense	0.43	**0.063**	0.03	0.849	Wnt-Repressed	*SLC2A9*	0.25	1.14E−01	48,453	2	9
in vivo	*ENSG00000224660*	*SH3BP5-AS1*	antisense	0.57	**0.018**	0.45	0.177	Wnt-Repressed	*SH3BP5*	− 0.36	2.44E−02	87,183	0	6
in vitro	*ENSG00000233930*	*KRTAP5-AS1*	antisense	− 0.09	0.794	− 0.50	**0.036**	Wnt-Activated	*DUSP8*	0.55	2.69E−04	*566*	7	2
in vitro	*XLOC_033478*	*XLOC_033478*	novel_lncRNAs	− 0.28	0.794	− 0.85	**0.009**	Wnt-Activated	*ID2*	0.28	8.11E−02	797,501	NA	NA
in vitro	*XLOC_005971*	*XLOC_005971*	novel_lncRNAs	0.58	0.834	− 0.82	**0.036**	Wnt-Activated	*NSL1*	0.02	8.94E−01	68,636	NA	NA
in vitro	*ENSG00000249042*	*CTD-2015H6.3*	antisense	− 0.21	0.640	0.55	**0.015**	Wnt-Activated	*ZFYVE16*	0.46	3.21E−03	80,049	6	4
in vitro	*ENSG00000246889*	*AP000487.5*	antisense	− 0.73	0.682	0.66	**0.001**	Wnt-Activated	*CTTN*	− 0.24	1.36E−01	*83*	8	3
in vitro	*XLOC_036743*	*XLOC_036743*	novel_lncRNAs	− 0.49	0.729	0.48	**0.066**	Wnt-Repressed	*LGALSL*	0.30	5.87E−02	78,230	NA	NA
in vitro	*ENSG00000264301*	*LINC01444*	lincRNA	− 0.43	0.908	0.76	**0.070**	Wnt-Repressed	*RNMT*	− 0.07	6.58E−01	1,243,807	2	0
in vitro	*ENSG00000215256*	*DHRS4-AS1*	antisense	0.28	0.569	0.63	**0.070**	Wnt-Activated	*DHRS4L2*	0.29	6.64E−02	18,964	2	5
in vitro	*ENSG00000224046*	*AC005076.5*	antisense	0.36	0.132	0.85	**0.001**	Wnt-Activated	*DMTF1*	− 0.38	1.64E−02	*58*	2	5

^a^log2FC: the enrichment/depletion of sgRNAs targeting a lncRNA, calculated based on log2 transformed fold change of read counts of the second best sgRNA targeting the lncRNA. Positive FC means sgRNA targeting increased cell growth in the screen; negative FC means sgRNA targeting decreased cell growth in the screen

^b^FDR, false discovery rate, calculated based on the fold change of all sgRNAs targeting the lncRNA compared to the non-targeting controls; FDR < 10% is highlighted in bold (see Methods)

^c^Correlation (nearest PCG): Spearman correlation coefficient of Wnt-regulated lncRNA with its nearest PCG in response to Wnt inhibition in the orthotopic HPAF-II cancer model

^d^lncRNA-PCG distance (bp): the distance in base pair between the TSS of Wnt-regulated lncRNA and its nearest PCG; distance less than 1 kb is highlighted in italic, as the PCG may be suppressed by sgRNA targeting the lncRNA

^e^Upregulated No. cancers: number of TCGA cancer types the lncRNA is upregulated, as determined by differential expression (FDR < 5%) between tumors and their paired normal samples

^f^Downregulated No. cancers: number of TCGA cancer types the lncRNA is downregulated, as determined by differential expression (FDR < 5%) between tumors and their paired normal samples

We found that the four Wnt-regulated lncRNA loci that were hits in both screens were essential for HPAF-II cancer cell growth (Fig. 4b, c). For example, 3 out of 5 sgRNAs targeting *LINC00263* were depleted in both screens, suggesting that it was an essential lncRNA for HPAF-II growth both in vivo and in vitro (Fig. 4d). Interestingly, *LINC00263* has previously been reported to be a cell type-specific lncRNA essential for the growth of U87 cells but not K562, HeLa, or MCF7 cells [45]. Twenty-one Wnt-regulated lncRNA loci were hits only in the in vivo screen and would not have been identified in an in vitro screen. Of these, 2 lncRNAs can promote cancer cell growth, while 19 lncRNAs appear to have suppressive effects on cell proliferation in vivo. For example, 4 sgRNAs targeting *ABHD11-AS1* were only enriched at the end of the in vivo, but not the in vitro screen (Fig. 4e). Among the 9 Wnt-regulated lncRNA loci that were hits only in the in vitro screen, we found 3 of them promoted, while 6 suppressed HPAF-II proliferation in culture. For example, all 5 sgRNAs targeting *AP000487.1* were enriched at the end of the in vitro screen; however, none of the 5 sgRNAs showed significant change after the in vivo screen (Fig. 4f). This suggests that *AP000487.1* may have tumor suppressive function only in vitro. Taken together, using CRISPRi screens both in vivo and in vitro, we identified Wnt-regulated lncRNA loci that modify HPAF-II growth in a context-dependent manner. It also suggests that a subset lncRNA loci identified in vitro may not have important functions in vivo.

To further validate the CRISPRi screen results, we focused on *LINC00263*, which was an essential lncRNA for HPAF-II cell growth both in vivo and in vitro (Fig. 4d). We cloned the top two sgRNAs targeting *LINC00263* into doxycycline-inducible lentiviral sgRNA vectors. We demonstrated that these sgRNAs can suppress *LINC00263* expression by ~ 80% in the CRISPRi system (Fig. 4g) and knocking down *LINC00263* reduced HPAF-II cell growth in vitro (Fig. 4h). Interestingly, we found that knocking down *LINC00263* also reduced the expression of its nearest protein-coding gene stearoyl-CoA desaturase (*SCD*) (Fig. 4g), similar to what was reported in U87 cells [45]. To test if *SCD* regulates the growth of HPAF-II cells, we next targeted the TSS of *SCD* using CRISPRi with two independent sgRNAs. Knockdown of *SCD* reduced *SCD* mRNA abundance (Additional file 1: Fig. S8) and inhibited HPAF-II cell growth similar to that observed after knockdown of *LINC00263* (Fig. 4i). However, sgRNAs targeting the TSS of *SCD* did not reduce the expression of *LINC00263* (Additional file 1: Fig. S8). Based on these results, we hypothesize that *LINC00263* is essential for HPAF-II cell growth through *cis*-regulation of *SCD*.

## Discussion

LncRNAs play important roles in diverse biological processes. Here we present a systematic study to identify and functionally assess lncRNAs regulated by Wnt signaling. Using an orthotopic Wnt-addicted pancreatic cancer model treated with a potent and effective PORCN inhibitor, we identified 1503 lncRNAs regulated by Wnt signaling in vivo. Many of these lncRNAs were also dysregulated in different cancer types and may function through gene networks that contribute to the pathogenesis of cancers. Our eQTL-lncRNA interactions’ analysis identified Wnt-regulated lncRNAs that may regulate nearby protein-coding genes. Using CRISPRi screens, we found that 34 Wnt-regulated lncRNAs could modify cell growth in a context-dependent manner with a higher hit rate in the in vivo model. This pipeline for lncRNA discovery and functional validation may be broadly applicable.

We previously reported that Wnt-regulated protein-coding genes were more robustly regulated in an orthotopic model than in cultured cells. We find that this holds true for lncRNAs as well. More than twice as many lncRNAs responded to Wnt inhibition in the in vivo xenografts than in cells cultured in vitro. These differences in the number and magnitude of gene expression changes will be influenced by a variety of local and experimental factors including tumor microenvironment, culture conditions, doubling times in different environments, local nutrients versus culture medium ingredients, the presence of stromal and other host cells, and variations in extracellular matrix. Overall, our findings are consistent with the large body of literature showing that the expression of genes is regulated by interaction with the relevant environment [72].

Cancer cells show differential dependencies on protein-coding genes for their growth and survival in vivo versus in vitro [51, 70, 73, 74]. Our CRISPRi screen results indicate that cancer cells also have different requirements for lncRNAs when grown in vivo vs in vitro conditions. Multiple lncRNAs exhibit different phenotypes when studied in cell culture compared to animal knock-out models and in vivo systems [75–79]. Our results highlight the importance of studying lncRNAs in vivo with the relevant microenvironment in order to better understand their functions in cancer pathogenesis. This has implications for the identification of lncRNAs as potential therapeutic targets for cancer treatment. For instance, it has been shown that drugs identified through high-throughput screening of cell culture in vitro have limited success in patient care [80, 81]. The same might be true for drugs identified to target lncRNAs.

Despite the large number of lncRNAs annotated in the human genome [8, 9], only a very small fraction of them have been either validated or characterized at a functional level. This is due to the complex nature of the lncRNA loci and a prior lack of tools to study them at a large scale [63, 75]. In recent years, CRISPR screens have been shown to be an efficient and specific approach to investigate lncRNA functions genome-wide in cultured cells [45, 82–85]. In this study, we perform a CRISPRi screen not only in cultured cells, but also in xenograft tumors to assess the ability of 1503 Wnt-regulated lncRNAs to influence cancer cell proliferation. Validating this approach, among the 4 Wnt-regulated lncRNAs that we found to be functional both in vivo and in vitro, 3 were identified to promote cell growth in prior CRISPRi screens [45]. Furthermore, consistent with what has been reported for genome-wide lncRNA CRISPRi screens in cell lines [45], 1% (13/1503) of the Wnt-regulated lncRNAs in our in vitro screen modified cancer cell growth. Notably, our in vivo CRISPRi screen identified twice as many Wnt-regulated lncRNAs (25/1503) that had a functional effect on cancer cell growth. Twenty-one Wnt-regulated lncRNAs had functional effects on cancer cell growth only in the xenograft model and would not have been identified in an in vitro screen, demonstrating the value of studying lncRNA functions in vivo. This is also demonstrated in a recent study that an in vivo system is essential for understanding the biological role of a human lncRNA in metabolic regulation that cannot be recapitulated in vitro [79].

The CRISPR-based approach can produce different results than those based on RNA interference. *LINC00176*, found in our screen as a functional Wnt-regulated lncRNA locus, has also been identified in four other publications. Two groups used different CRISPR approaches (paired-sgRNAs [84] or sgRNA targeting splice site [85]) and found, as we did, that *LINC00176* has a tumor-suppressive effect in vivo. Two additional studies used RNA interference and concluded, conversely, that *LINC00176* has a pro-proliferative role in ovarian and hepatocellular carcinoma cell lines [86, 87]. These differences could be due to variations in cell type or experimental approach, as RNA interference is known to suffer from significantly more off-target effects compared to the CRISPR approach and is less effective for targeting nuclear lncRNAs [88, 89]. Together, the comparisons reported here further support the identification of Wnt-regulated lncRNA loci that can modify cancer cell growth and the importance of choosing a loss-of-function strategy to characterize lncRNAs.

Nevertheless, there are some limitations to using CRISPRi to target lncRNAs. First, recruiting dCas9-KRAB to the TSS of a lncRNA can suppress the transcriptional activity and local regulatory sequence (enhancer) of the lncRNA locus; second, it results in decreased production of the lncRNA transcript, inhibiting potential *cis* or *trans* function of the lncRNA transcript [45]. Both the repressive effect on chromatin and the lack of lncRNA transcripts can cause biological consequences that cannot be differentiated by CRISPRi knock-down alone. In addition, due to the nature of CRISPRi screens, it is not possible to know how effectively each of the designed sgRNAs suppresses the expression of the target lncRNA, so there is the possibility of false negatives. As with all screens, additional studies are needed to verify the results and to further dissect how the Wnt-regulated lncRNA loci identified in our screen regulate cell proliferation.

GWAS studies have identified thousands of common genetic variants that are associated with complex traits and diseases, but 90% of these fall into noncoding regions of the genome [90]. This has made it difficult to dissect the underlying molecular mechanisms. eQTLs that co-localize with GWAS SNPs suggest the effect of the SNPs on diseases and traits is mediated by changes in gene expression. lncRNAs overlapping with these GWAS-associated *cis*-eQTL SNPs are potential candidates to explain the underlying mechanisms of risk loci because lncRNAs can be important *cis* regulators of nearby genes [54–56]. When we mapped Wnt-regulated lncRNAs-mRNA pairs linked by eQTL SNPs using the annotation from FANTOM5 [8], we found previously unappreciated regulatory effects of Wnt-regulated lncRNAs in disease. For example, Wnt-regulated lncRNA *LINC00339* was linked to *CDC42* through five eQTL SNPs, suggesting the *LINC00339* locus may regulate the expression of *CDC42*. Supporting this, knocking-down *LINC00339* expression has been reported to increase *CDC42* expression [91]. Consistent with the importance of Wnt regulation, *LINC00339* and its linked gene *CDC42* are involved in both endometriosis and bone metabolism [91, 92], two Wnt-regulated biological processes [93, 94]. Thus, identifying eQTL-linked Wnt-regulated lncRNA-PCG pairs helps to prioritize the potential *cis*-regulatory targets of Wnt-regulated lncRNAs. Further integrating the disease risk information based on GWAS SNPs co-localizing with eQTL, the Wnt-regulated lncRNA-PCG pairs may help explain the underlying mechanisms of risk loci in the context of disease, which is potentially affected by Wnt signaling. We also examined the eQTL-linked Wnt-regulated lncRNA-PCG pair co-expressions both globally in FANTOM5 dataset across cell types and tissues and specifically in TCGA pancreatic cancer. We observed that more lncRNA-PCG pairs were significantly co-expressed in the pancreatic cancer (1070 pairs) than globally across cell types and tissues (555 pairs), which could be due to the tissue-specific functions of lncRNAs [95].

Although the 1503 Wnt-regulated lncRNAs were discovered in the orthotopic *RNF43-*mutant pancreatic cancer xenograft model, many of them were also dysregulated in different types of cancers in TCGA (Additional file 1: Fig. S2A). A total of 248 Wnt-regulated lncRNAs were exclusively upregulated or downregulated across different cancer types (Additional file 2: Table S1). This suggests the fundamental role of Wnt-regulated lncRNAs in cancer pathogenesis in a broader context beyond Wnt-addicted pancreatic cancer. For example, *CCAT1*, identified as a Wnt-activated lncRNA, was also upregulated in 9 cancer types, including pancreatic cancer (Additional file 2: Table S1, Additional file 3: Table S2). Our CRISPRi screens indicated that it is an essential lncRNA both in vivo and in vitro (Table 1). This suggests that *CCAT1* is a Wnt-activated lncRNA with oncogenic function, which is consistent with previous studies showing that *CCAT1* can promote the progression of different types of cancers [96–98]. Integrating Wnt-regulated lncRNAs with their expression profiles in TCGA and CRISPRi functional screens can better distinguish their oncogenic or tumor suppressive functions in cancer pathogenesis.

## Conclusions

This study comprehensively identified 1503 lncRNAs regulated by Wnt signaling in vivo and determined their wider roles in other cancers. We found more than twice as many lncRNAs responded to Wnt inhibition in the in vivo xenografts than in cells cultured in vitro. With CRISPRi screens both in vivo and in vitro, we found two fold (21/1503) as many Wnt-regulated lncRNAs have functional effects on cell growth only in vivo, suggesting the importance of studying lncRNA function with relevant microenvironment. Thus, this study provides a valuable resource of functional Wnt-regulated lncRNAs in vivo. It also establishes a framework for integrating orthogonal transcriptomics dataset with functional CRISPRi screening which can be broadly adapted for systematic discovery, functional annotation, and validation of lncRNAs in vivo.

## Supplementary information

**Additional file 1: Supplementary figure file containing Figs. S1-S8**. **Fig. S1.** ETC-159 inhibits Wnt/β-catenin signaling. **Fig. S2.** Wnt-regulated lncRNAs are dysregulated in TCGA cancers. **Fig. S3.** Subset of Wnt-regulated lncRNAs co-express with its nearest PCG in the same TAD. **Fig. S4.** Clusters are enriched for genes dysregulated in different cancers. **Fig. S5.** Validation of CRISPRi system for gene suppression. **Fig. S6.** A high correlation of sgRNA counts between independent experimental replicates in CRISPRi screens. **Fig. S7.** CRISPRi screens are able to identify important positive controls as gene hits. **Fig. S8.** Knockdown of *SCD* with CRISPRi reduce *SCD* mRNA abundance, but not the expression of *LINC00263*.

**Additional file 2: Table S1.** 1503 Wnt-regulated lncRNAs.

**Additional file 3: Table S2.** 78 Wnt-regulated lncRNAs that are significantly up- or down-regulated in pancreatic cancer.

**Additional file 4: Table S3.** Wnt-regulated lncRNA-PCG pairs linked by eQTL SNPs involving 602 Wnt-regulated lncRNAs.

**Additional file 5: Table S4.** Wnt-regulated lncRNA-PCG pairs linked by eQTL SNPs that colocalize with cancer GWAS loci.

**Additional file 6: Table S5.** Influence of Wnt inhibition on the co-expression of lncRNA-PCG pairs.

**Additional file 7: Table S6**. Time-series clustering results and Gene Ontology functional enrichment analysis for significant biological processes of each cluster.

**Additional file 8: Table S7**. sgRNA libraries used in CRISPRi screens.

**Additional file 9: Table S8**. Normalized sgRNA read counts from in vivo and in vitro CRISPRi screens.

**Additional file 10: Table S9**. Gene level screen results of Wnt-regulated lncRNAs and protein-coding gens that have TSS within 1 kb of the TSS of Wnt-regulated lncRNAs from in vivo and in vitro CRISPRi screens.

## Data Availability

The RNA-seq data for HPAF-II orthotopic, subcutaneous, and in vitro model are available at the NCBI’s Gene Expression Omnibus repository, under the accession numbers GSE118041, GSE118179, GSE118190 [26]. The full reference transcriptome GTF file and time-course RNA-seq reads quantification are available in the figshare repository at 10.6084/m9.figshare.13012535 [99]. Detailed results for the Wnt-regulated lncRNAs can be found in Additional file 2: Table S1. The TAD data from the PANC-1 cell line was downloaded from the 3D Genome Browser [38]. The gene expression data for all the TCGA cancers are available in the UCSC Xena platform [39]. The eQTL-linked lncRNA PCG pairs were downloaded from FANTOM5 annotation eQTL_linked_lncRNA_mRNA_pair [8]. The gene expression profiles of 1829 FANTOM5 samples were downloaded from the expression atlas FANTOM_CAT.expression_atlas.gene.lv3_robust.rle_cpm curated by FANTOM5 [8].

## References

[1] Brown CJ, Ballabio A, Rupert JL, Lafreniere RG, Grompe M, Tonlorenzi R (1991). A gene from the region of the human X inactivation Centre is expressed exclusively from the inactive X chromosome. Nature..

[2] Brannan CI, Dees EC, Ingram RS, Tilghman SM (1990). The product of the H19 gene may function as an RNA. Mol Cell Biol.

[3] Huarte M (2015). The emerging role of lncRNAs in cancer. Nat Med.

[4] Huarte M, Guttman M, Feldser D, Garber M, Koziol MJ, Kenzelmann-Broz D (2010). A large intergenic noncoding RNA induced by p53 mediates global gene repression in the p53 response. Cell..

[5] Trimarchi T, Bilal E, Ntziachristos P, Fabbri G, Dalla-Favera R, Tsirigos A (2014). Genome-wide mapping and characterization of Notch-regulated long noncoding RNAs in acute leukemia. Cell..

[6] Yuan J-H, Yang F, Wang F, Ma J-Z, Guo Y-J, Tao Q-F (2014). A long noncoding RNA activated by TGF-β promotes the invasion-metastasis cascade in hepatocellular carcinoma. Cancer Cell.

[7] Schmitt AM, Chang HY (2016). Long noncoding RNAs in cancer pathways. Cancer Cell.

[8] Hon C-C, Ramilowski JA, Harshbarger J, Bertin N, Rackham OJL, Gough J (2017). An atlas of human long non-coding RNAs with accurate 5′ ends. Nature..

[9] Iyer MK, Niknafs YS, Malik R, Singhal U, Sahu A, Hosono Y (2015). The landscape of long noncoding RNAs in the human transcriptome. Nat Genet.

[10] Nusse R, Clevers H (2017). Wnt/β-catenin signaling, disease, and emerging therapeutic modalities. Cell..

[11] Polakis P (2012). Wnt signaling in cancer. Cold Spring Harb Perspect Biol.

[12] Zhan T, Rindtorff N, Boutros M (2017). Wnt signaling in cancer. Oncogene..

[13] Zhong Z, Virshup DM (2020). Wnt signaling and drug resistance in Cancer. Mol Pharmacol.

[14] Jiang X, Hao H-X, Growney JD, Woolfenden S, Bottiglio C, Ng N (2013). Inactivating mutations of RNF43 confer Wnt dependency in pancreatic ductal adenocarcinoma. Proc Natl Acad Sci U S A.

[15] Koo B-K, Spit M, Jordens I, Low TY, Stange DE, van de Wetering M (2012). Tumour suppressor RNF43 is a stem-cell E3 ligase that induces endocytosis of Wnt receptors. Nature..

[16] Seshagiri S, Stawiski EW, Durinck S, Modrusan Z, Storm EE, Conboy CB (2012). Recurrent R-spondin fusions in colon cancer. Nature..

[17] Bailey P, Chang DK, Nones K, Johns AL, Patch A-M, Gingras M-C (2016). Genomic analyses identify molecular subtypes of pancreatic cancer. Nature..

[18] Cancer Genome Atlas Research Network. Electronic address: andrew_aguirre@dfci.harvard.edu, Cancer Genome Atlas Research Network. Integrated genomic characterization of pancreatic ductal adenocarcinoma. Cancer Cell. 2017;32:185–203.e13. 10.1016/j.ccell.2017.07.007 PMC5964983 28810144

[19] Giannakis M, Hodis E, Jasmine Mu X, Yamauchi M, Rosenbluh J, Cibulskis K (2014). RNF43 is frequently mutated in colorectal and endometrial cancers. Nat Genet.

[20] Waddell N, Pajic M, Patch A-M, Chang DK, Kassahn KS, Bailey P (2015). Whole genomes redefine the mutational landscape of pancreatic cancer. Nature..

[21] Madan B, Virshup DM (2015). Targeting Wnts at the source--new mechanisms, new biomarkers, new drugs. Mol Cancer Ther.

[22] Willert K, Brown JD, Danenberg E, Duncan AW, Weissman IL, Reya T (2003). Wnt proteins are lipid-modified and can act as stem cell growth factors. Nature..

[23] Chen B, Dodge ME, Tang W, Lu J, Ma Z, Fan C-W (2009). Small molecule-mediated disruption of Wnt-dependent signaling in tissue regeneration and cancer. Nat Chem Biol.

[24] Madan B, Ke Z, Harmston N, Ho SY, Frois AO, Alam J (2016). Wnt addiction of genetically defined cancers reversed by PORCN inhibition. Oncogene..

[25] Ng M, Tan DSP, Subbiah V, Weekes CD, Teneggi V, Diermayr V (2017). First-in-human phase 1 study of ETC-159 an oral PORCN inhbitor in patients with advanced solid tumours. J Clin Oncol.

[26] Madan B, Harmston N, Nallan G, Montoya A, Faull P, Petretto E (2018). Temporal dynamics of Wnt-dependent transcriptome reveal an oncogenic Wnt/MYC/ribosome axis. J Clin Invest.

[27] Kawasaki Y, Komiya M, Matsumura K, Negishi L, Suda S, Okuno M (2016). MYU, a target lncRNA for Wnt/c-Myc signaling, mediates induction of CDK6 to promote cell cycle progression. Cell Rep.

[28] Giakountis A, Moulos P, Zarkou V, Oikonomou C, Harokopos V, Hatzigeorgiou AG (2016). A positive regulatory loop between a Wnt-regulated non-coding RNA and ASCL2 controls intestinal stem cell fate. Cell Rep.

[29] Taniue K, Kurimoto A, Takeda Y, Nagashima T, Okada-Hatakeyama M, Katou Y (2016). ASBEL-TCF3 complex is required for the tumorigenicity of colorectal cancer cells. Proc Natl Acad Sci U S A.

[30] Conway T, Wazny J, Bromage A, Tymms M, Sooraj D, Williams ED (2012). Xenome--a tool for classifying reads from xenograft samples. Bioinformatics..

[31] Kim D, Pertea G, Trapnell C, Pimentel H, Kelley R, Salzberg SL (2013). TopHat2: accurate alignment of transcriptomes in the presence of insertions, deletions and gene fusions. Genome Biol.

[32] Trapnell C, Williams BA, Pertea G, Mortazavi A, Kwan G, van Baren MJ (2010). Transcript assembly and quantification by RNA-Seq reveals unannotated transcripts and isoform switching during cell differentiation. Nat Biotechnol.

[33] Anders S, Pyl PT, Huber W (2015). HTSeq--a Python framework to work with high-throughput sequencing data. Bioinformatics..

[34] Wang L, Park HJ, Dasari S, Wang S, Kocher J-P, Li W (2013). CPAT: Coding-Potential Assessment Tool using an alignment-free logistic regression model. Nucleic Acids Res.

[35] Kong L, Zhang Y, Ye Z-Q, Liu X-Q, Zhao S-Q, Wei L (2007). CPC: assess the protein-coding potential of transcripts using sequence features and support vector machine. Nucleic Acids Res.

[36] Chen J, Shishkin AA, Zhu X, Kadri S, Maza I, Guttman M (2016). Evolutionary analysis across mammals reveals distinct classes of long non-coding RNAs. Genome Biol.

[37] Love MI, Huber W, Anders S (2014). Moderated estimation of fold change and dispersion for RNA-seq data with DESeq2. Genome Biol.

[38] Wang Y, Song F, Zhang B, Zhang L, Xu J, Kuang D (2018). The 3D Genome Browser: a web-based browser for visualizing 3D genome organization and long-range chromatin interactions. Genome Biol.

[39] Goldman MJ, Craft B, Hastie M, Repečka K, McDade F, Kamath A (2020). Visualizing and interpreting cancer genomics data via the Xena platform. Nat Biotechnol.

[40] Zhao S, Li C-I, Guo Y, Sheng Q, Shyr Y (2018). RnaSeqSampleSize: real data based sample size estimation for RNA sequencing. BMC Bioinformatics..

[41] Hensman J, Lawrence ND, Rattray M (2013). Hierarchical Bayesian modelling of gene expression time series across irregularly sampled replicates and clusters. BMC Bioinformatics.

[42] Hensman J, Rattray M, Lawrence ND (2015). Fast nonparametric clustering of structured time-series. IEEE Trans Pattern Anal Mach Intell.

[43] Reimand J, Arak T, Adler P, Kolberg L, Reisberg S, Peterson H (2016). g:Profiler-a web server for functional interpretation of gene lists (2016 update). Nucleic Acids Res.

[44] Horlbeck MA, Gilbert LA, Villalta JE, Adamson B, Pak RA, Chen Y, et al. Compact and highly active next-generation libraries for CRISPR-mediated gene repression and activation. Elife [Internet]. 2016;5. Available from: 10.7554/eLife.19760. 10.7554/eLife.19760 PMC5094855 27661255

[45] Liu SJ, Horlbeck MA, Cho SW, Birk HS, Malatesta M, He D, et al. CRISPRi-based genome-scale identification of functional long noncoding RNA loci in human cells. Science. 2017;355. 10.1126/science.aah7111 PMC5394926 27980086

[46] Aubrey BJ, Kelly GL, Kueh AJ, Brennan MS, O’Connor L, Milla L (2015). An inducible lentiviral guide RNA platform enables the identification of tumor-essential genes and tumor-promoting mutations in vivo. Cell Rep.

[47] Adamson B, Norman TM, Jost M, Cho MY, Nuñez JK, Chen Y (2016). A multiplexed single-cell CRISPR screening platform enables systematic dissection of the unfolded protein response. Cell.

[48] Li W, Xu H, Xiao T, Cong L, Love MI, Zhang F (2014). MAGeCK enables robust identification of essential genes from genome-scale CRISPR/Cas9 knockout screens. Genome Biol.

[49] Idris M, Harmston N, Petretto E, Madan B, Virshup DM (2019). Broad regulation of gene isoform expression by Wnt signaling in cancer. RNA..

[50] Gabay M, Li Y, Felsher DW. MYC activation is a hallmark of cancer initiation and maintenance. Cold Spring Harb Perspect Med [Internet]. 2014;4. Available from: 10.1101/cshperspect.a014241. 10.1101/cshperspect.a014241 PMC4031954 24890832

[51] Miller TE, Liau BB, Wallace LC, Morton AR, Xie Q, Dixit D (2017). Transcription elongation factors represent in vivo cancer dependencies in glioblastoma. Nature..

[52] Muir A, Vander Heiden MG (2018). The nutrient environment affects therapy. Science..

[53] Whiteside TL (2008). The tumor microenvironment and its role in promoting tumor growth. Oncogene..

[54] Engreitz JM, Haines JE, Perez EM, Munson G, Chen J, Kane M (2016). Local regulation of gene expression by lncRNA promoters, transcription and splicing. Nature..

[55] Gil N, Ulitsky I (2020). Regulation of gene expression by cis-acting long non-coding RNAs. Nat Rev Genet.

[56] Luo S, Lu JY, Liu L, Yin Y, Chen C, Han X (2016). Divergent lncRNAs regulate gene expression and lineage differentiation in pluripotent cells. Cell Stem Cell.

[57] Dixon JR, Selvaraj S, Yue F, Kim A, Li Y, Shen Y (2012). Topological domains in mammalian genomes identified by analysis of chromatin interactions. Nature..

[58] GTEx Consortium, Laboratory, Data Analysis &Coordinating Center (LDACC)—Analysis Working Group, Statistical Methods groups—Analysis Working Group, Enhancing GTEx (eGTEx) groups, NIH Common Fund, NIH/NCI, et al. Genetic effects on gene expression across human tissues. Nature. 2017;550:204–213. 10.1038/nature24277 PMC5776756 29022597

[59] Gao P, Xia J-H, Sipeky C, Dong X-M, Zhang Q, Yang Y (2018). Biology and clinical implications of the 19q13 aggressive prostate cancer susceptibility locus. Cell.

[60] Li Q, Seo J-H, Stranger B, McKenna A, Pe’er I, Laframboise T (2013). Integrative eQTL-based analyses reveal the biology of breast cancer risk loci. Cell..

[61] Tan JY, Smith AAT, Ferreira da Silva M, Matthey-Doret C, Rueedi R, Sönmez R (2017). cis-Acting complex-trait-associated lincRNA expression correlates with modulation of chromosomal architecture. Cell Rep.

[62] Guttman M, Donaghey J, Carey BW, Garber M, Grenier JK, Munson G (2011). lincRNAs act in the circuitry controlling pluripotency and differentiation. Nature..

[63] Kopp F, Mendell JT (2018). Functional classification and experimental dissection of long noncoding RNAs. Cell..

[64] Rotival M, Petretto E (2014). Leveraging gene co-expression networks to pinpoint the regulation of complex traits and disease, with a focus on cardiovascular traits. Brief Funct Genomics.

[65] Zhang J, Sui S, Wu H, Zhang J, Zhang X, Xu S (2019). The transcriptional landscape of lncRNAs reveals the oncogenic function of LINC00511 in ER-negative breast cancer. Cell Death Dis.

[66] Holtzhausen A, Zhao F, Evans KS, Tsutsui M, Orabona C, Tyler DS (2015). Melanoma-derived Wnt5a promotes local dendritic-cell expression of IDO and immunotolerance: opportunities for pharmacologic enhancement of immunotherapy. Cancer Immunol Res.

[67] Spranger S, Bao R, Gajewski TF (2015). Melanoma-intrinsic β-catenin signalling prevents anti-tumour immunity. Nature..

[68] Cai L, Chang H, Fang Y, Li G (2016). A comprehensive characterization of the function of LincRNAs in transcriptional regulation through long-range chromatin interactions. Sci Rep.

[69] Gilbert LA, Larson MH, Morsut L, Liu Z, Brar GA, Torres SE (2013). CRISPR-mediated modular RNA-guided regulation of transcription in eukaryotes. Cell..

[70] Zhong Z, Sepramaniam S, Chew XH, Wood K, Lee MA, Madan B (2019). PORCN inhibition synergizes with PI3K/mTOR inhibition in Wnt-addicted cancers. Oncogene..

[71] Gilbert LA, Horlbeck MA, Adamson B, Villalta JE, Chen Y, Whitehead EH (2014). Genome-scale CRISPR-mediated control of gene repression and activation. Cell..

[72] Killion JJ, Radinsky R, Fidler IJ (1998). Orthotopic models are necessary to predict therapy of transplantable tumors in mice. Cancer Metastasis Rev.

[73] Possik PA, Müller J, Gerlach C, Kenski JCN, Huang X, Shahrabi A (2014). Parallel in vivo and in vitro melanoma RNAi dropout screens reveal synthetic lethality between hypoxia and DNA damage response inhibition. Cell Rep.

[74] Yau EH, Kummetha IR, Lichinchi G, Tang R, Zhang Y, Rana TM (2017). Genome-wide CRISPR screen for essential cell growth mediators in mutant KRAS colorectal cancers. Cancer Res.

[75] Bassett AR, Akhtar A, Barlow DP, Bird AP, Brockdorff N, Duboule D (2014). Considerations when investigating lncRNA function in vivo. Elife..

[76] Goudarzi M, Berg K, Pieper LM, Schier AF. Individual long non-coding RNAs have no overt functions in zebrafish embryogenesis, viability and fertility. Elife [Internet]. 2019;8. Available from: 10.7554/eLife.40815. 10.7554/eLife.40815 PMC6347452 30620332

[77] Han X, Luo S, Peng G, Lu JY, Cui G, Liu L (2018). Mouse knockout models reveal largely dispensable but context-dependent functions of lncRNAs during development. J Mol Cell Biol.

[78] Kohtz JD (2014). Long non-coding RNAs learn the importance of being in vivo. Front Genet.

[79] Ruan X, Li P, Chen Y, Shi Y, Pirooznia M, Seifuddin F (2020). In vivo functional analysis of non-conserved human lncRNAs associated with cardiometabolic traits. Nat Commun.

[80] Letai A (2017). Functional precision cancer medicine-moving beyond pure genomics. Nat Med.

[81] Sharma SV, Haber DA, Settleman J (2010). Cell line-based platforms to evaluate the therapeutic efficacy of candidate anticancer agents. Nat Rev Cancer.

[82] Esposito R, Bosch N, Lanzós A, Polidori T, Pulido-Quetglas C, Johnson R (2019). Hacking the Cancer genome: profiling therapeutically actionable long non-coding RNAs using CRISPR-Cas9 screening. Cancer Cell.

[83] Joung J, Engreitz JM, Konermann S, Abudayyeh OO, Verdine VK, Aguet F (2017). Genome-scale activation screen identifies a lncRNA locus regulating a gene neighbourhood. Nature..

[84] Zhu S, Li W, Liu J, Chen C-H, Liao Q, Xu P (2016). Genome-scale deletion screening of human long non-coding RNAs using a paired-guide RNA CRISPR-Cas9 library. Nat Biotechnol.

[85] Liu Y, Cao Z, Wang Y, Guo Y, Xu P, Yuan P (2018). Genome-wide screening for functional long noncoding RNAs in human cells by Cas9 targeting of splice sites. Nat Biotechnol.

[86] Dai L, Niu J, Feng Y (2020). Knockdown of long non-coding RNA LINC00176 suppresses ovarian cancer progression by BCL3-mediated down-regulation of ceruloplasmin. J Cell Mol Med.

[87] Tran DDH, Kessler C, Niehus SE, Mahnkopf M, Koch A, Tamura T (2018). Myc target gene, long intergenic noncoding RNA, Linc00176 in hepatocellular carcinoma regulates cell cycle and cell survival by titrating tumor suppressor microRNAs. Oncogene..

[88] Smith I, Greenside PG, Natoli T, Lahr DL, Wadden D, Tirosh I (2017). Evaluation of RNAi and CRISPR technologies by large-scale gene expression profiling in the Connectivity Map. PLoS Biol.

[89] Stojic L, Lun ATL, Mangei J, Mascalchi P, Quarantotti V, Barr AR (2018). Specificity of RNAi, LNA and CRISPRi as loss-of-function methods in transcriptional analysis. Nucleic Acids Res.

[90] Hindorff LA, Sethupathy P, Junkins HA, Ramos EM, Mehta JP, Collins FS (2009). Potential etiologic and functional implications of genome-wide association loci for human diseases and traits. Proc Natl Acad Sci U S A.

[91] Chen X-F, Zhu D-L, Yang M, Hu W-X, Duan Y-Y, Lu B-J (2018). An osteoporosis risk SNP at 1p36.12 acts as an allele-specific enhancer to modulate LINC00339 expression via long-range loop formation. Am J Hum Genet.

[92] Powell JE, Fung JN, Shakhbazov K, Sapkota Y, Cloonan N, Hemani G (2016). Endometriosis risk alleles at 1p36.12 act through inverse regulation of CDC42 and LINC00339. Hum Mol Genet.

[93] Krishnan V, Bryant HU, Macdougald OA (2006). Regulation of bone mass by Wnt signaling. J Clin Invest.

[94] Wang Y, Hanifi-Moghaddam P, Hanekamp EE, Kloosterboer HJ, Franken P, Veldscholte J (2009). Progesterone inhibition of Wnt/beta-catenin signaling in normal endometrium and endometrial cancer. Clin Cancer Res.

[95] Cabili MN, Trapnell C, Goff L, Koziol M, Tazon-Vega B, Regev A (2011). Integrative annotation of human large intergenic noncoding RNAs reveals global properties and specific subclasses. Genes Dev.

[96] Jiang Y, Jiang Y-Y, Xie J-J, Mayakonda A, Hazawa M, Chen L (2018). Co-activation of super-enhancer-driven CCAT1 by TP63 and SOX2 promotes squamous cancer progression. Nat Commun.

[97] Xiang J-F, Yin Q-F, Chen T, Zhang Y, Zhang X-O, Wu Z (2014). Human colorectal cancer-specific CCAT1-L lncRNA regulates long-range chromatin interactions at the MYC locus. Cell Res.

[98] Zhang E, Han L, Yin D, He X, Hong L, Si X (2017). H3K27 acetylation activated-long non-coding RNA CCAT1 affects cell proliferation and migration by regulating SPRY4 and HOXB13 expression in esophageal squamous cell carcinoma. Nucleic Acids Res.

[99] Liu S. Wnt-regulated lncRNA discovery enhanced by in vivo identification and CRISPRi functional validation. figshare. 2020. 10.6084/m9.figshare.13012505. 10.1186/s13073-020-00788-5 PMC7580003 33092630

