# Supplementary material for: Wnt-regulated lncRNA discovery enhanced by in vivo identification and CRISPRi functional validation
Source: Genome Med. 2020 Oct 22;12:89. doi: 10.1186/s13073-020-00788-5 (PMC7580003; doi:10.1186/s13073-020-00788-5)
Supplement: Supplementary file 1 — Additional file 1: Supplementary figure file containing Figs. S1-S8. Fig. S1. ETC-159 inhibits Wnt/β-catenin signaling. Fig. S2. Wnt-regulated lncRNAs are dysregulated in TCGA cancers. Fig. S3. Subset of Wnt-regulated lncRNAs co-express with its nearest PCG in the same TAD. Fig. S4. Clusters are enriched for genes dysregulated in different cancers. Fig. S5. Validation of CRISPRi system for gene suppression. Fig. S6. A high correlation of sgRNA counts between independent experimental replicates in CRISPRi screens. Fig. S7. CRISPRi screens are able to identify important positive controls as gene hits. Fig. S8. Knockdown of SCD with CRISPRi reduce SCD mRNA abundance, but not the expression of LINC00263. [file 13073_2020_788_MOESM1_ESM.pdf]

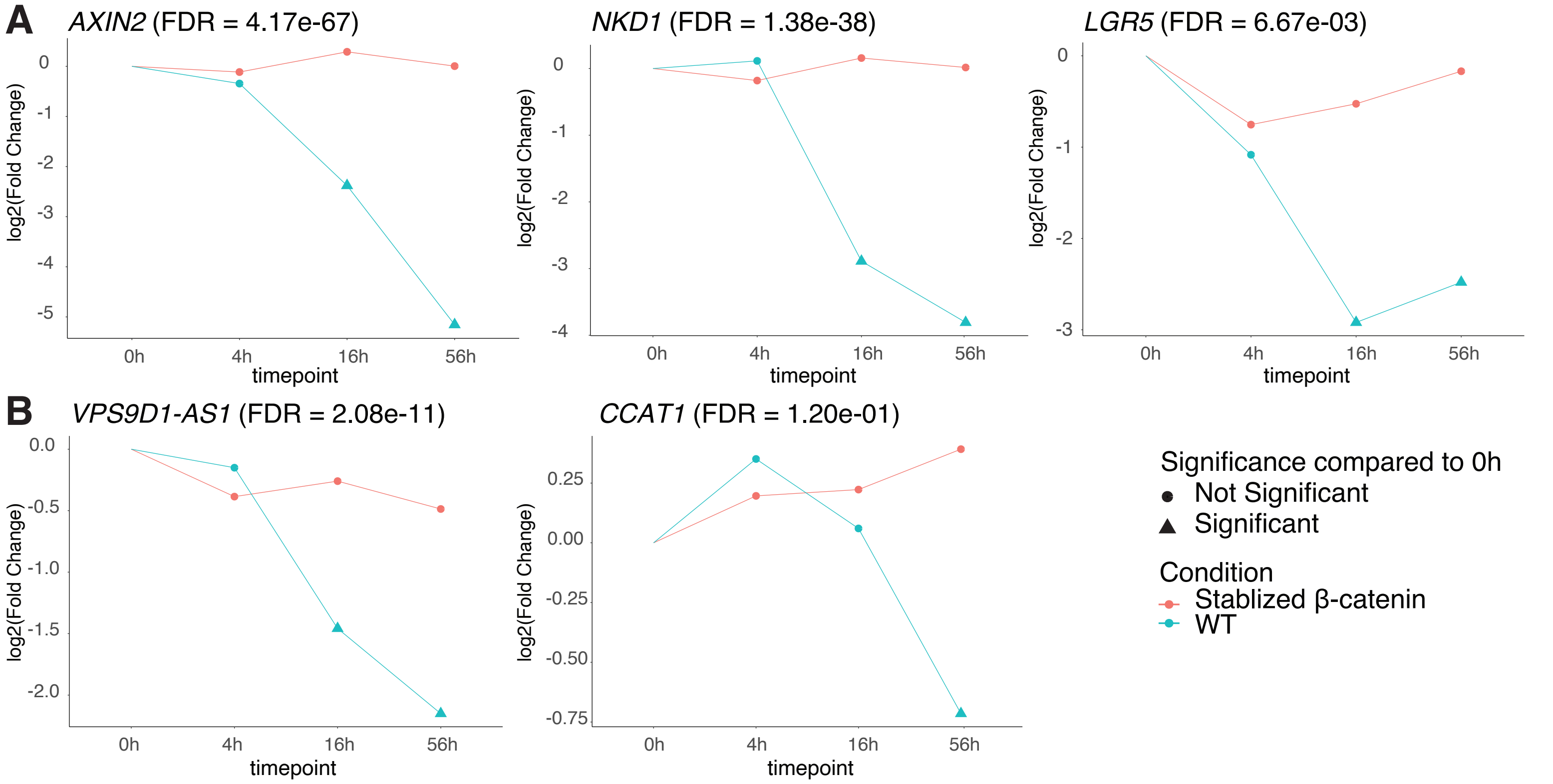

Fig. S1 ETC-159 inhibits Wnt/β-catenin signaling. (A) To demonstrate the specificity of ETC-159, we generated HPAF-II cells that express a stabilized non-phosphorylatable form of β-catenin. HPAF-II orthotopic xenografts were generated through orthotopic injection of HPAF-II cells or HPAF-II cells that express stabilized β-catenin. Mice with established orthotopic xenografts were treated with 37.5 mg/kg ETC-159 twice daily by oral gavage. The tumors were harvested at 4 hours, 16 hours and 56 hours after initial drug treatment for gene expression analysis. Wnt target genes (*AXIN2, NKD1, LGR5*) are significantly downregulated with ETC-159 treatment after 16 hours in HPAF-II cells; the inhibition effect can be rescued with the expression of stabilized β-catenin.
(B) Wnt-regulated lncRNAs *VPS9D1-AS1* and *CCAT1* are downregulated after ETC-159 treatment in WT HPAF-II cells but the inhibition effect is abolished in the HPAF-II cells with stabilized β-catenin.

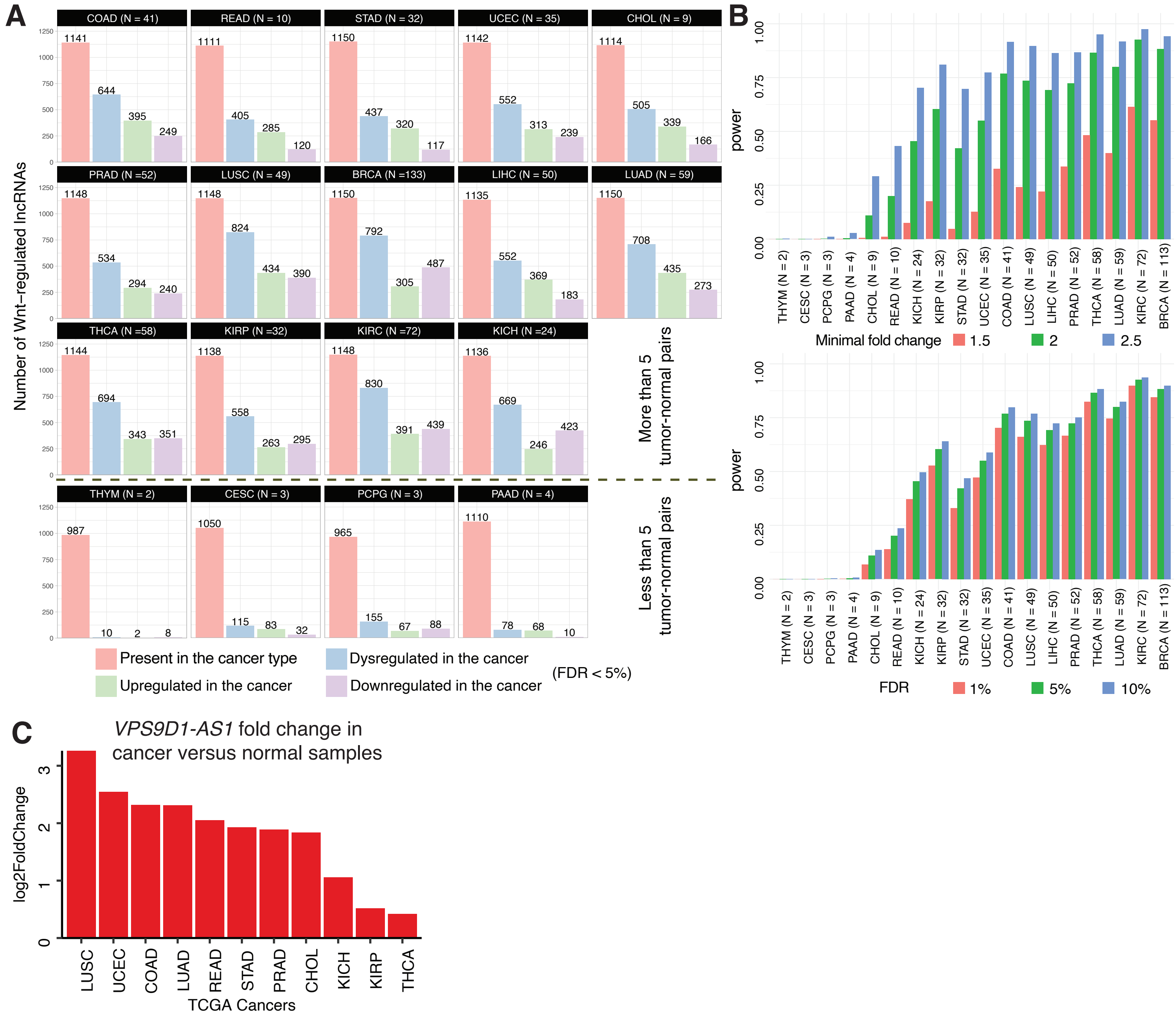

Fig. S2 Wnt-regulated lncRNAs are dysregulated in TCGA cancers. (A) Wnt-regulated lncRNAs are dysregulated in different types of cancers as determined by differential expression between tumors and their paired normal samples using the TCGA dataset. (B) Statistical power to find significantly differentially expressed genes between tumor and paired normal samples for different cancer types, at different minimal fold change and FDR. Cancers with less than 5 tumor-normal pairs have much lower statistical power (close to 0) to identify significantly dysregulated genes compared to cancers with more than 5 tumor-normal pairs. (C) *VPS9D-AS1* is upregulated in 11 different types of cancers.

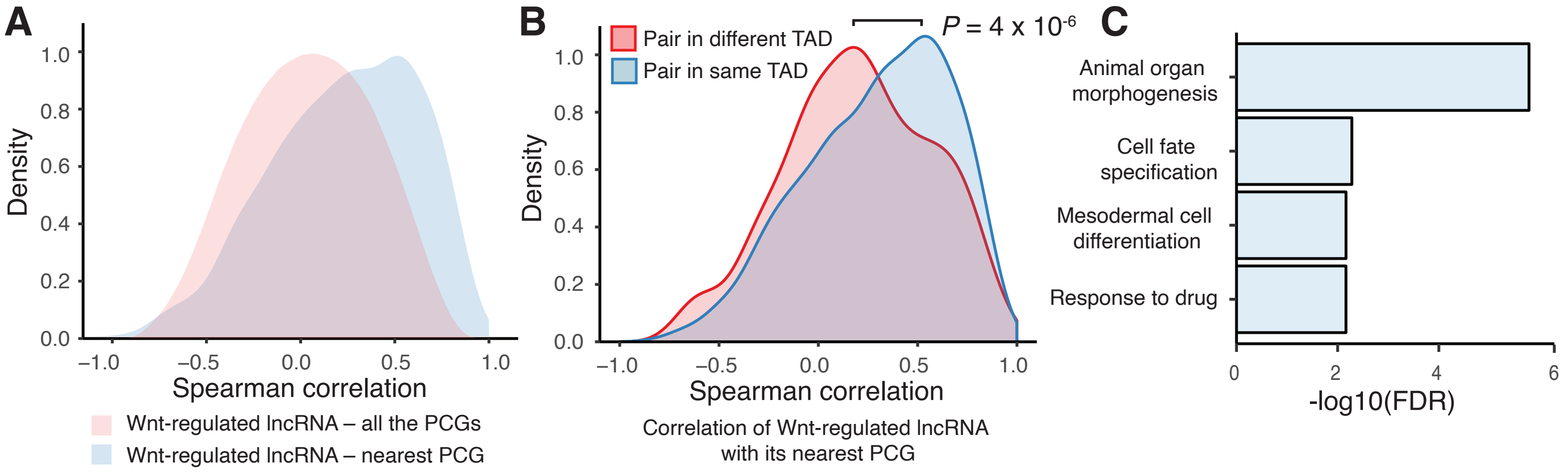

Fig. S3 Subset of Wnt-regulated lncRNAs co-express with its nearest PCG in the same TAD. (A) Wnt-regulated lncRNAs exhibit stronger co-expression with their nearest PCG to Wnt inhibition compared to their co-expression with all PCGs. (B) Wnt-regulated lncRNA–nearest PCG pairs within the same TAD exhibit stronger co-expression than the pairs in different TADs. *P* for significance was calculated by Mann–Whitney U test. (C) These highly co-expressed Wnt-regulated lncRNAs that are proximal to PCGs and co-localized within the same TAD, are likely to be involved in the same cellular processes of their neighbour PCGs, such as organ development and cell fate specification.

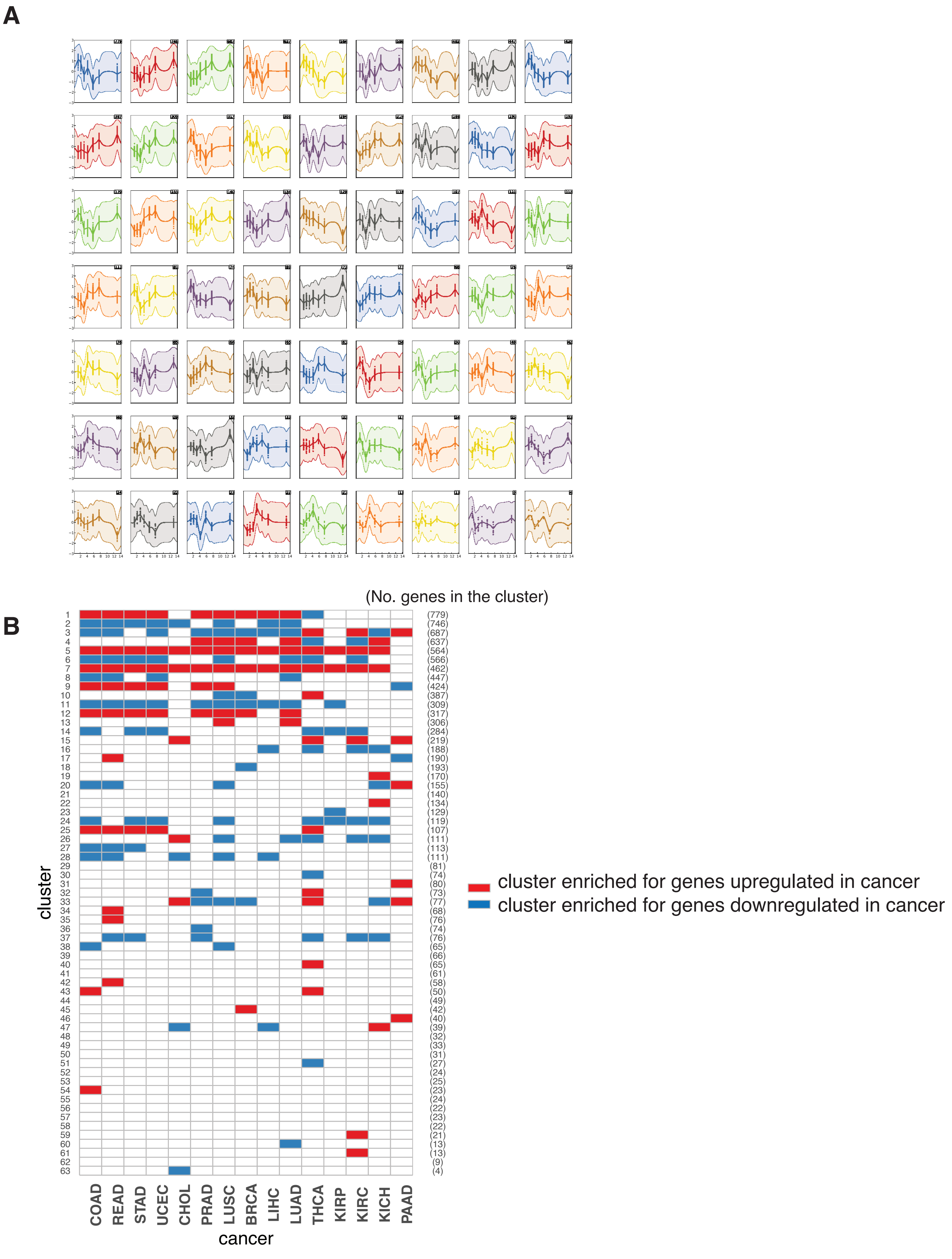

Fig. S4 Clusters are enriched for genes dysregulated in different cancers. (A) The Wnt-regulated lncRNAs and PCGs fall into 63 distinct clusters based on their pattern of expression change following Wnt inhibition. (B) 48 out of the 63 clusters are enriched (FDR < 5%) for genes dysregulated in at least one type of cancer.

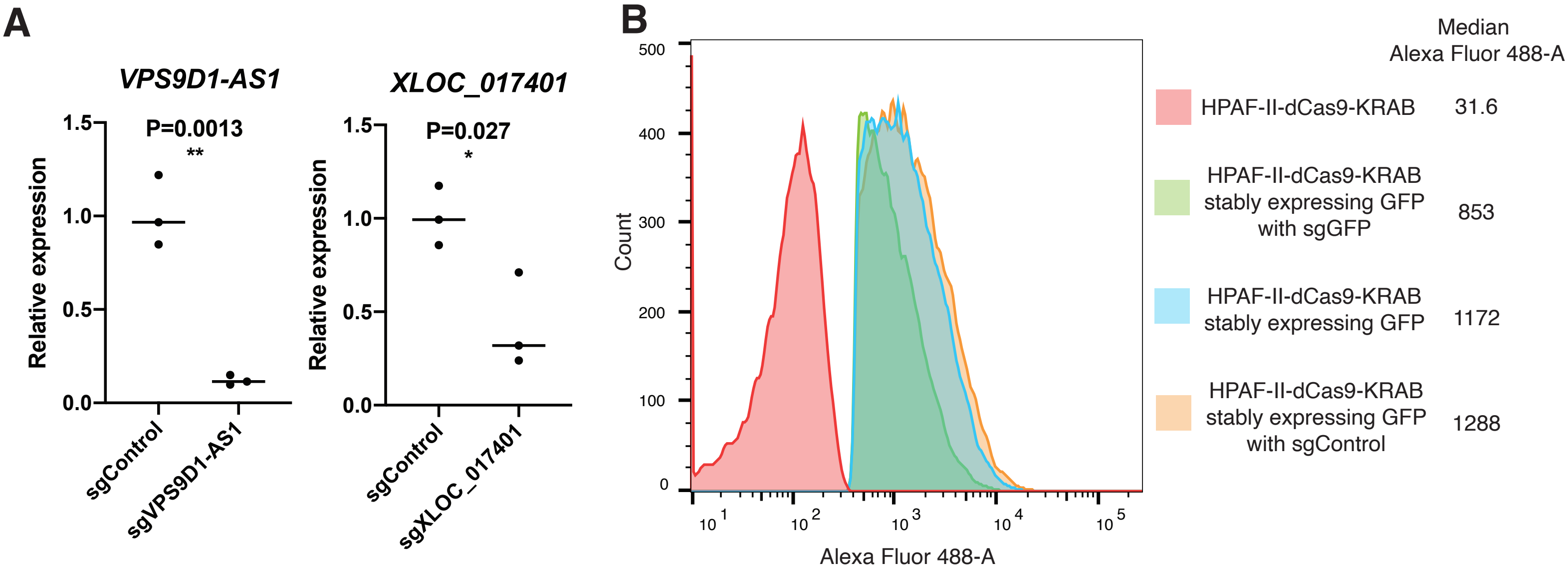

Fig. S5 Validation of CRISPRi system for gene suppression. (A) CRISPRi system can suppress *VPS9D1-AS1* and *XLOC_017401* expression. (B) A specific sgRNA is able to knock down GFP expression in HPAF-II-dCas9-KRAB cells that stably expressed GFP.

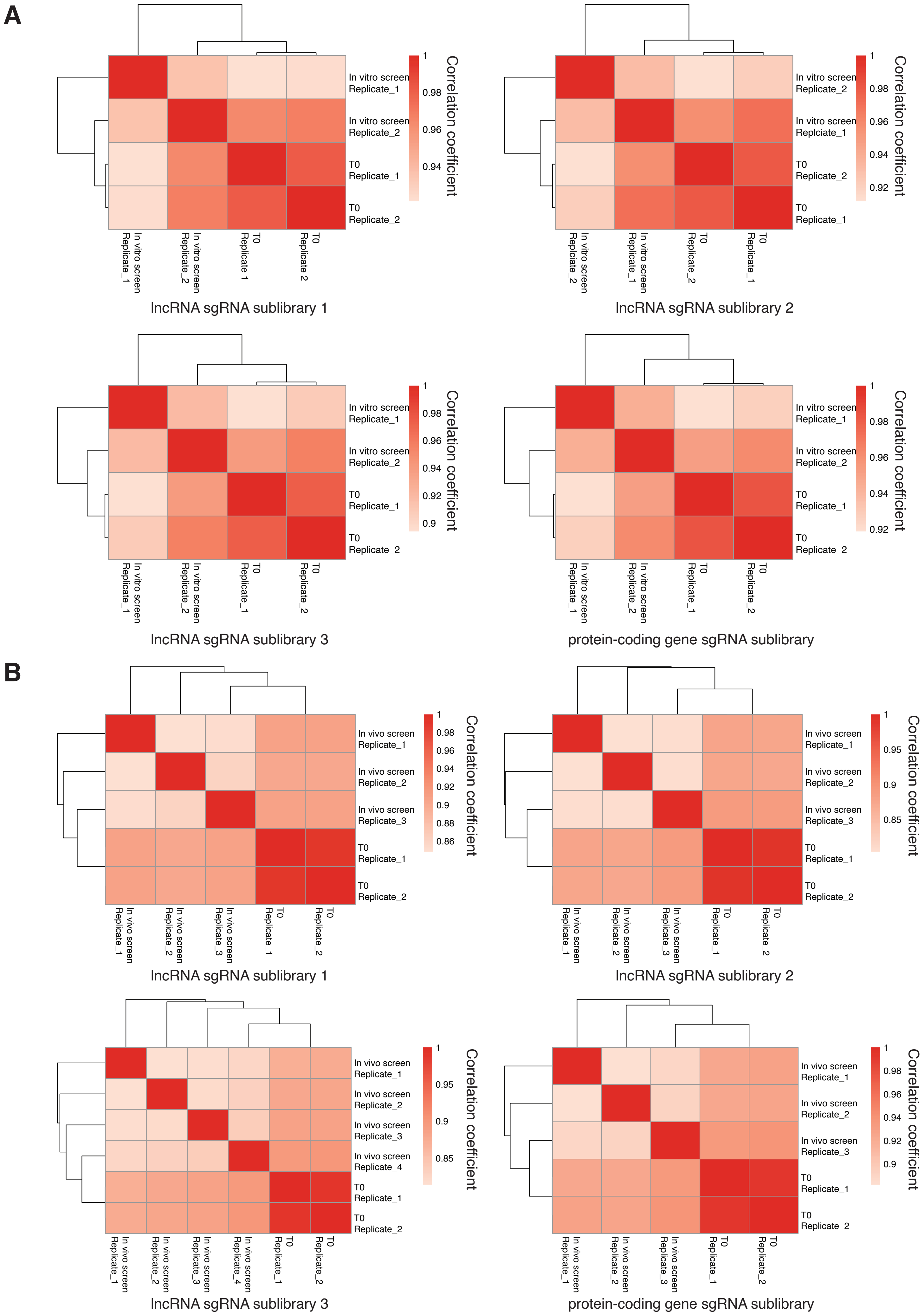

Fig. S6 A high correlation of sgRNA counts between independent experimental replicates in CRISPRi screens. (A) Correlation of sgRNA counts between experimental replicates in the *in vitro* screens. (B) Correlation of sgRNA counts between experimental replicates in the *in vivo* screens.

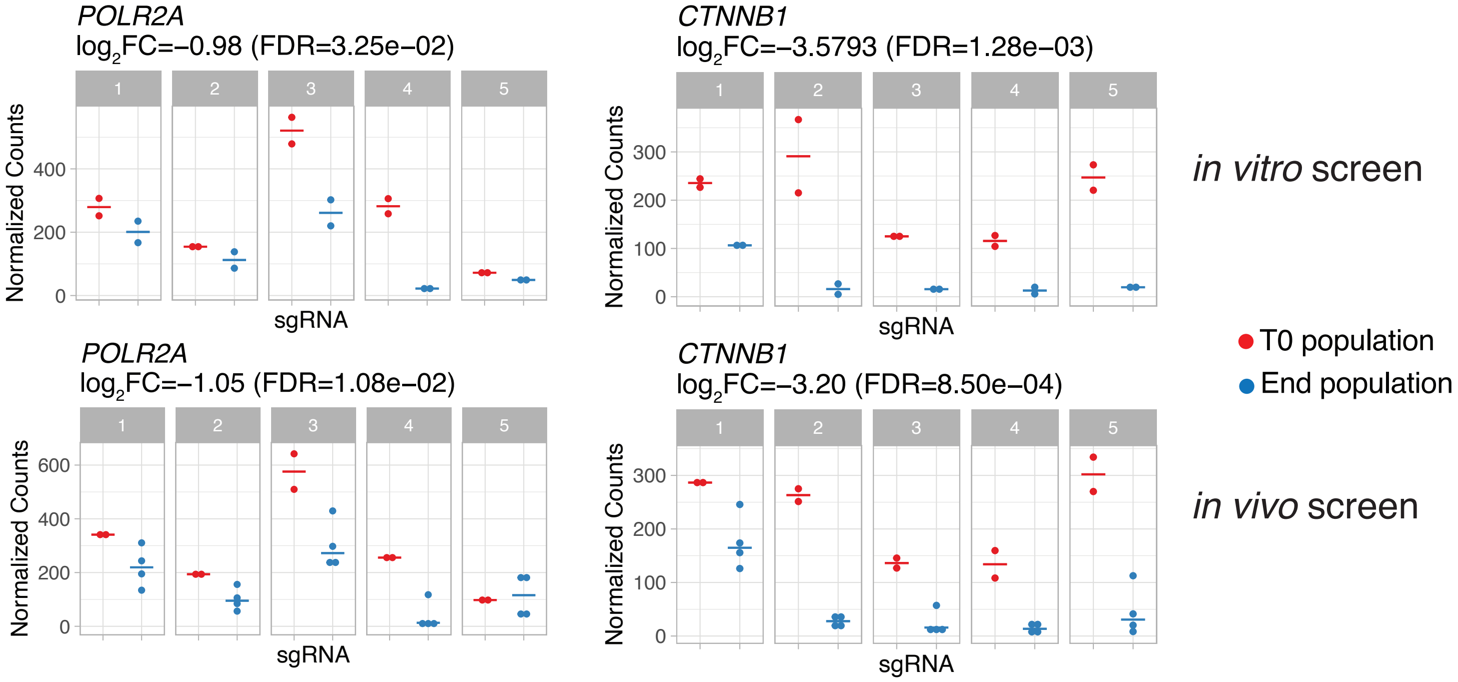

Fig. S7 CRISPRi screens are able to identify important positive controls as gene hits.

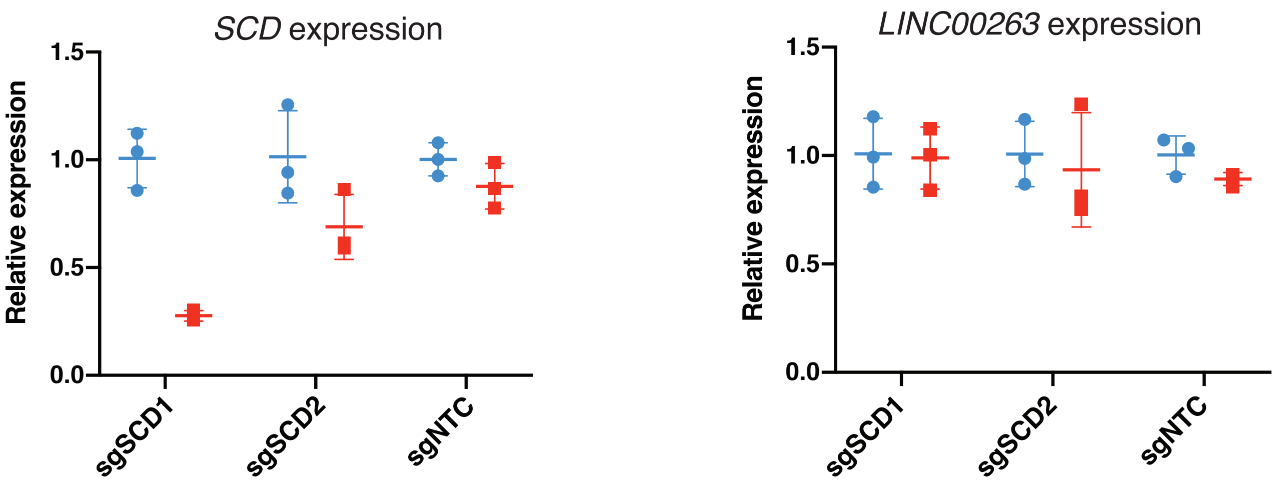

Fig. S8 Knockdown of SCD with CRISPRi reduce *SCD* mRNA abundance, but not the expression of *LINC00263*.
